# Different Aspects of Dominance Are Not Equivalent When Testing for Trade‐Offs in Ant Communities

**DOI:** 10.1002/ece3.72207

**Published:** 2025-09-21

**Authors:** Annika S. Nelson, Kailen A. Mooney

**Affiliations:** ^1^ Department of Biology Texas Christian University Fort Worth Texas USA; ^2^ Rocky Mountain Biological Laboratory Crested Butte Colorado USA; ^3^ Department of Ecology and Evolutionary Biology University of California at Irvine Irvine California USA

**Keywords:** ants, colonization, competition, discovery ability, dominance, trade‐offs

## Abstract

Differences in dominance are frequently invoked to explain the outcomes of competition. Yet, what it means to be dominant, and which traits underlie dominance, are poorly understood. Here, we sought to determine the relationships between multiple aspects of dominance, the potential for trade‐offs with discovery ability, and the traits associated with these patterns within a high elevation community of five ant taxa. We examined several common dominance metrics—behavioral dominance (winning aggressive encounters at both the individual and colony levels), numerical dominance (abundance and activity in baits and pitfall traps), and ecological dominance (high relative frequency in baits)—and found that individual‐ and colony‐level behavioral dominance was positively correlated, as were ecological and numerical dominance. However, colony‐level behavioral and numerical dominance were negatively correlated, and no other dominance metrics were associated. There was a dominance‐discovery trade‐off, as increased behavioral (but not numerical or ecological) dominance was associated with slower resource discovery. This trade‐off was likely driven by behaviorally dominant ants having larger body sizes and recruiting a greater biomass of workers to baits. In contrast, fast discoverers were more abundant in the environment (i.e., numerically dominant). Complementing our empirical study, a meta‐analysis of 54 responses from 21 studies showed that the association between dominance and discovery ability depended on the dominance metric. Whereas discovery ability was positively correlated with numerical dominance, its relationships with behavioral and ecological dominance were highly variable and not significantly different from zero. Overall, our empirical findings, in combination with the synthesis of past studies, demonstrate that different aspects of ant dominance are not equivalent. Yet, regardless of dominance type, there is little evidence that dominance‐discovery trade‐offs occur in most ant communities.

## Graphical abstract

Dominance hierarchies are often invoked to explain the outcomes of competition across ecological communities. However, there are a number of difficulties associated with quantifying dominance, making it is challenging to ultimately link dominance to community structure and species coexistence. Here we provide evidence, both empirically and through meta‐analysis, that different forms of dominance commonly measured in ant communities are not equivalent and have different relationships with the ability to discover food resources quickly. In doing so, we demonstrate the importance of better defining dominance and its underlying traits when assessing the outcomes of competition in ant communities and across systems.

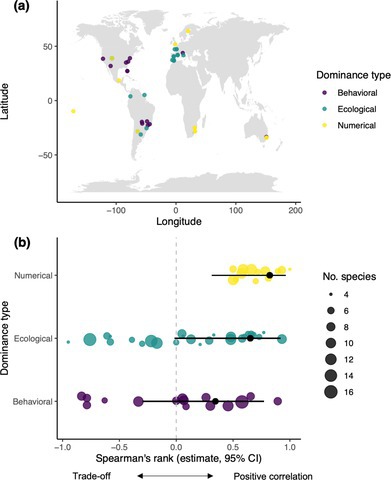

## 1 Introduction

Within populations and communities, organisms can often be ranked according to their ability to win conflicts and gain priority access to limiting resources (Tibbetts et al. 2022). Such dominance hierarchies are associated with a range of traits, including those linked to physiology, behavior, and reproduction, and may structure ecological communities (Wright et al. 2020; Princen et al. 2020; Milewski et al. 2022). Yet, there are multiple challenges associated with constructing dominance hierarchies. For instance, dominance can be conceptualized and quantified in different ways, leading to potentially conflicting findings that obscure the role of competition in structuring ecological communities (Levy et al. 2020).

Competition has been dubbed the “hallmark of ant ecology” (Hölldobler and Wilson 1990). The role of interspecific dominance hierarchies in shaping competition among ant species for food has been studied extensively, both within (Parr and Gibb 2010; Cerdá et al. 2013) and among communities (Sheard et al. 2020). One widely held belief is that trade‐offs between dominance and other traits determine the outcomes of competition and can potentially promote the coexistence of ant species. The most widely studied trade‐off in ant communities is that between dominance and resource discovery ability (Fellers 1987; Stanton et al. 2002), which is analogous to the competition‐colonization trade‐off. This trade‐off was first described in plants (Tilman 1994; Turnbull et al. 1999; Amarasekare 2003) and has since been demonstrated across many taxa, including birds (Rodríguez et al. 2007), insects (Stanton et al. 2002), and microbes (Smith et al. 2018). In ants, it occurs when specialized morphological, physiological, or behavioral traits that increase dominance reduce the ability to discover resources (Parr and Gibb 2010). However, evidence for this trade‐off in most ant communities is controversial (Parr and Gibb 2012; Sheard et al. 2020). A previous meta‐analysis of 18 datasets found a significantly positive, rather than negative, mean correlation between dominance and discovery ability of food (Parr and Gibb 2012). This could suggest that this trade‐off does not play a major role in structuring interactions among ants, but it is also possibly under‐reported due to inconsistencies in the ways in which dominance is defined and measured across studies (Stuble et al. 2017).

Ants are typically ranked according to one of several possible types of dominance—behavioral, numerical, or ecological dominance (Table 1; Parr and Gibb 2010). Behaviorally dominant ants excel in interference competition by deterring other species during encounters at food resources, either through superior aggressive behaviors (e.g., charging, biting, or spraying toxic chemicals) or recruitment abilities (Parr and Gibb 2010). Numerically dominant ants excel in exploitative competition for food as a result of occurring in the greatest numbers, frequency, or biomass in the environment (Andersen 1997; Bestelmeyer 2000; Parr 2008). Ecologically dominant ants have disproportionately high foraging success relative to their abundance in the environment (e.g., Cerdá et al. 1997; LeBrun 2005). It is likely that different sets of traits underlie these forms of dominance, allowing ants to be dominant in different ways (e.g., behaviorally but not numerically dominant; Lach 2005; Segev and Ziv 2012). However, because few studies have examined how behavioral, numerical, and ecological dominance are related (but see Stuble et al. 2017), it is unclear whether or when they are equivalent or how closely any metric is ultimately tied to resource discovery ability.

**TABLE 1:** Dominance and discovery metrics included in the meta‐analysis. Note that although this and other empirical studies calculated the same form of dominance in multiple ways (e.g., behavioral dominance at both the individual and colony levels), we included only one dominance calculation from each study (the one that was most commonly used across studies) to include in the meta‐analysis.

Dominance or discovery form	Definition	Metric(s)	Citations
Behavioral dominance	Having superior aggressive behaviors that deter other ants	Number/proportion of encounters won in baits (raw proportions or Colley scores); “colony‐level behavioral dominance”	This study; Fellers (1987), Holway (1999), Gibb and Hochuli (2004), Lebrun and Feener (2007), Santini et al. (2007), Feener et al. (2008), Lessard et al. (2009), Wiescher et al. (2011), Stuble et al. (2013), Sales et al. (2014), Camarota et al. (2018)
Numerical dominance	Occurring in the greatest numbers, frequency, or biomass	Number/proportion of baits occupied/monopolized	This study; Sarty et al. (2006), Parr and Gibb (2012), Antoniazzi et al. (2021), Chifflet and Calcaterra (2025)
Ecological dominance	Having the greatest foraging success relative to abundance in the environment	Number/proportion of baits occupied/monopolized, out of all pitfall traps where that species occurred	This study; Calcaterra et al. (2016)
		Number/proportion of baits monopolized, out of all baits where that species occurred	Castracani et al. (2014), Achury et al. (2020), Sheard et al. (2020), Klunk and Pie (2021), Chifflet and Calcaterra (2025)
Discovery ability	Having the ability to discover resources quickly due to high foraging efficiency or abundance	Number/proportion of all baits discovered first	Sarty et al. (2006), Lebrun and Feener (2007), Antoniazzi et al. (2021), Chifflet and Calcaterra (2025)
		Number/proportion of baits discovered first, out of all baits where present	Achury et al. (2020), Calcaterra et al. (2016), Camarota et al. (2018), Castracani et al. (2014), Fellers (1987), Klunk and Pie (2021), Parr and Gibb (2012), Sales et al. (2014), Santini et al. (2007), Sheard et al. (2020), Wiescher et al. (2011)
		Time to discover/recruit to baits	This study; Holway (1999), Gibb and Hochuli (2004), Feener et al. (2008), Stuble et al. (2013)
		Residuals of the number of baits discovered, regressed against abundance in pitfall traps	Lessard et al. (2009)

A trait‐based approach could help clarify the relationships between different forms of dominance and their association with the ability to discover food (Ferzoco and McCauley 2023; Jelley and Moreau 2023). We expect large body or colony size to be associated with behavioral dominance, as these traits can heighten ant aggression and improve success in interference competition (Retana et al. 2015). Similarly, we predict that numerically dominant ants should have larger colonies, greater colony abundance and activity, or both, as this may increase ant abundance in baits. However, numerical dominance is not necessarily associated with worker body size, and behavioral dominance is not necessarily associated with colony abundance. Because behavioral and numerical dominance likely share some but not all underlying traits, the association between these dominance metrics should depend on the relative degree to which shared versus unique traits contribute to overall dominance scores. Whereas some dominance‐associated traits may reduce resource discovery ability (e.g., when larger bodied ants cannot maneuver around obstacles to discover resources as quickly; Farji‐Brener 2004; Farji‐Brener et al. 2004), others may improve it (e.g., when ants with greater colony sizes or abundances discover resources faster simply by chance; Segev and Ziv 2012).

Multiple studies have found evidence for the dominance–discovery trade‐off when focused on behavioral dominance (e.g., Feener et al. 2008; Stuble et al. 2013), and this is the metric with which the trade‐off was originally demonstrated (Fellers 1987). Yet, in other cases, behavioral dominance and discovery ability are positively correlated, such as when the ants that arrive first at a food source are able to actively defend and maintain control of it (“discovery‐defense strategy”; Camarota et al. 2018; Antoniazzi et al. 2021). Studies that have tested for the trade‐off based on numerical or ecological dominance have often also found a positive correlation (e.g., Parr and Gibb 2012; Sheard et al. 2020). Yet, to our knowledge, no prior empirical or meta‐analytical study has explicitly compared the relationships between multiple forms of dominance and discovery ability, making it difficult to determine whether different dominance metrics are equivalent.

Here, we examined the relationships between behavioral, numerical, and ecological dominance and tested for trade‐offs with the ability to discover food in a high‐elevation (2940 m) ant community. Specifically, we asked: (1) Are behavioral, numerical, and ecological dominance correlated? (2) Do any forms of dominance trade off with food discovery ability? and (3) What are the traits associated with the dominance‐discovery trade‐off? To compare the findings from this study with those previously reported in the literature, we conducted a meta‐analysis of 54 responses from 21 studies reporting on the association between multiple forms of dominance and discovery ability. In doing so, this study demonstrates that different aspects of dominance are not equivalent. Nonetheless, regardless of dominance type, dominance‐discovery trade‐offs are uncommon, suggesting that other mechanisms are more important for structuring most ant communities.

## 2 Materials and Methods

### 2.1 Study Sites and Natural History

**FIGURE 1:**
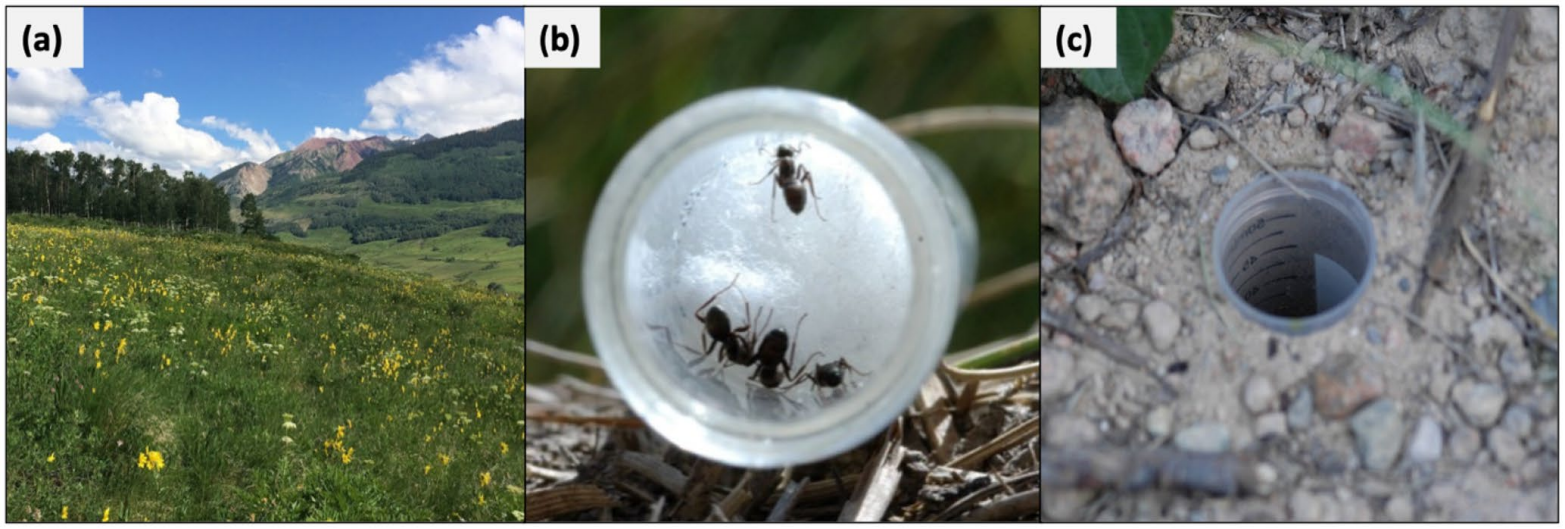
Photographs of (a) one of the 16 subalpine meadows in Gothic, CO, where sampling of ground‐nesting ant communities occurred, (b) *Formica podzolica* ants recruited to a 20% honey bait, and (c) a pitfall trap used to sample the ant communities.

This study was conducted in June–August 2016–2017 near the Rocky Mountain Biological Laboratory (RMBL) in Gothic, CO (38.96° N, 106.99° W; Figure 1a). We worked in 16 subalpine meadows at a mean elevation of 2940 m (± 29.8 SD; site coordinates in Appendix S1: Table S1). Sites were distributed across a 4‐km radius, separated by a minimum of 50 m. Five ground‐dwelling ant groups within three subfamilies commonly occur in these sites: *Camponotus* spp., *Formica podzolica* , *Formica rufa* species group (subfamily Formicinae), *Myrmica* spp. (subfamily Myrmicinae), and *Tapinoma sessile* (subfamily Dolichoderinae; Mooney et al. 2016; Nelson, Symanski, et al. 2019; Nelson, Pratt, et al. 2019; Nelson et al. 2020). Some of these groups consist of multiple species (e.g., the *Formica rufa* species group), as species within groups are difficult to distinguish in the field and are functionally very similar (Nelson & Mooney, *unpublished data*). While there is surely variation in traits among species within the same group, this variation is small relative to that among groups. We acknowledge that grouping species reduces statistical power, but an advantage of this approach is that it allows us to account for phylogenetic non‐independence among closely related species with similar traits. Because sugar‐rich liquids (i.e., hemipteran‐produced honeydew and extrafloral nectar) are main components of the diets of all taxa in this study (Nelson and Mooney 2022), we measured competition for sugar‐rich baits. Although we also assessed ant competition for other resource types (e.g., lipids, proteins, and various amino acids) in additional surveys within the same sites, results were qualitatively similar, and for simplicity we thus report results from sugar baits only.

### 2.2 Data Collection

#### 2.2.1 Behavioral Dominance

We evaluated ant behavioral dominance (i.e., success in interference competition) at both the individual and colony levels. For individual‐level behavioral dominance, we used a simple assay to measure aggression between pairs of taxa in one‐on‐one encounters (adapted from Suarez et al. 2002). Although individual‐level aggression is most often used to study nestmate recognition (rather than interspecific competition), it also allows for a high level of control in studies of ant interactions with competitors. Yet, most ants do not forage alone, and foragers can behave differently in groups. For instance, some species that lose one‐on‐one interactions can still succeed in displacing competitors due to cooperative fighting (Buczkowski and Bennett 2007). For this reason, we also measured ant behavioral dominance at the colony level using baits (described below), which is often, but not always, positively correlated with individual‐level behavior (Wittman and Gotelli 2011). While this approach provides more ecological realism, one‐on‐one assays allow for more detailed observations, particularly of interactions among species that do not frequently co‐occur within baits. The two approaches thus yield complementary measures of behavioral dominance.

In 11 of the 16 sites in 2016 and 2017 (listed in Appendix S1), we quantified individual‐level behavioral dominance by opportunistically collecting pairs of ants foraging on sugar‐rich resources (i.e., extrafloral nectar or hemipteran‐produced honeydew) from within approximately 5 m of each other. We placed the two workers (of different taxa) together in a 15‐mL plastic vial and recorded their interactions for 5 min. Interactions were scored following a 4‐point scale, in order of increasing aggression: 1 = touch (ants did not run away or attack each other), 2 = avoid (one or both ants quickly ran in opposite directions), 3 = aggression (brief behaviors, < 2 s, including lunging, biting, or using chemical defenses), and 4 = fight (prolonged aggression, > 2 s, often causing death or dismemberment; Suarez et al. 2002). We recorded the number of interactions of each score during the encounter. A total of 87 trials were conducted, with 4–19 replicates of each unique pair of taxa, depending on worker availability. Each pair was tested in a minimum of two sites.

We then constructed matrices of the outcomes of these assays (Appendix S1: Table S2). Similar to Suarez et al. (2002), we considered an ant to win the interaction if it exhibited a greater number of aggressive behaviors (scores > 2) or fewer avoidance behaviors (score = 2) than its opponent during the assay. Ants that exhibited equal numbers of aggressive or avoidance behaviors or only ever touched (score = 1) were considered to have tied. Trials in which no interactions occurred (i.e., ants never came into contact) were excluded from analyses. Winners of each assay were assigned a score of 1, losers were assigned a score of 0, and ties were included as 1/2 points for both taxa. Behavioral dominance was calculated as the total number of wins and ties each taxon had against all other taxa, divided by the total number of assays (raw scores in Appendix S1: Table S2). Because scores can be biased by differences in sample size, we corrected them using Colley's bias‐free method (Appendix S1: Table S2), which takes into account not only the number of wins and losses, but also the dominance scores of each species' competitors (Lebrun and Feener 2007; Stuble et al. 2013).

We measured colony‐level behavioral dominance by observing the outcomes of interactions among workers at baits (Lebrun and Feener 2007; Feener et al. 2008; Stuble et al. 2013). In each of the 16 sites in 2016, baits were deployed at 10 stations on a 5 × 2 grid, with each spaced 10 m apart along the grid to avoid repeatedly sampling ants from the same colony (diagram in Appendix S1: Figure S1). At each station, we deployed one bait consisting of a 15 mL plastic centrifuge tube filled with 10 mL of a 20% solution by mass of forest honey [honey produced by bees ( *Apis mellifera* ) foraging on hemipteran honeydew (Langnese Forest Honey, Bargteheide, Germany)] (Figure 1b). Baits were fitted with a cotton wick and deployed between 8:00 and 9:00 a.m. We recorded the number and identity of all ants visiting the baits at least once every 30 min for the first 1.5 h. Depending on ant activity, baits were left in the field for up to 5 days and checked at least once per day. While baits were primarily observed in the mornings (between 7:30 a.m. and 12:00 p.m.), we also observed them in the afternoons and evenings (between 12:00 and 9:30 p.m.) within six of the sites. Because observations spanned a range of times, any differences in foraging activity throughout the day should have been captured.

An ant was considered to win an interaction if it replaced another taxon previously occupying that bait. Taxa were considered to tie if they repeatedly co‐occurred at a bait across subsequent observations. Although we were unable to directly observe most instances of aggression between ants, as multiple baits were deployed over an extended period, replacements of taxa at baits were presumably the direct result of interference competition, as few sugar‐rich resources are left unattended. When multiple replacements occurred at the same bait across observations, we included each replacement as a separate interaction in the analyses. Winners of each interaction were assigned a score of 1, losers were assigned a score of 0, and ties were included as 1/2 points for both taxa (Appendix S1: Table S2). Raw colony‐level behavioral dominance scores were then calculated as the total number of wins and ties of each taxon, divided by the total number of interactions (scores in Appendix S1: Table S2). However, because these scores could be biased by not only differences in sample size among taxa but also the non‐independence of repeated interactions within the same bait, we calculated the proportion of interactions taxa won against each competitor within each bait. We then obtained estimated marginal mean dominance scores from a linear mixed effects model (LMER) that predicted the proportion of interactions won per bait based on ant taxon and competitor identity, with site and bait within site as random effects. These corrected scores were thus obtained for each ant species by controlling for other potential sources of variation, including non‐independence among multiple observations from the same baits.

#### 2.2.2 Numerical Dominance

We estimated numerical dominance, or dominance due to occurring at a high abundance and activity in the environment, by measuring ant occurrence in baits (sampling described above) and in pitfall traps. We placed 10 pitfall traps 1 m to the right of each bait along the same 2 × 5 grid in each site (Appendix S1: Figure S1). Traps were 50 mL plastic centrifuge tubes with 2.75 cm diameters, filled with soapy water and placed flush with the ground surface (Figure 1c). They were deployed 24 h after baiting ended to prevent ant recruitment to baits from biasing pitfall trap data. After 26–72 h (sampling longer when weather was less favorable for ant foraging), we recorded the identity of ants present in each trap. Numerical dominance was calculated as the mean proportion of baits occupied per site, as well as the mean proportion of pitfall traps occupied per site. We used these two approaches to estimate ant colony abundance and activity, as it was not possible to visually locate colonies due to dense vegetation (Nelson, Symanski, et al. 2019). Although trapping and baiting were conducted at different frequencies and durations across sites, all traps and baits within a site were sampled at the same times. We accounted for site‐level differences in sampling effort when calculating numerical dominance by extracting estimated marginal mean numerical dominance scores for each ant species from a linear mixed effect model (LMER) that included sampling effort as a covariate and site as a random effect (additional details below and in Appendix S1).

#### 2.2.3 Ecological Dominance

Ecological dominance, or the ability to access a disproportionately high number of resources relative to abundance in the environment, was measured based on the frequency of ant occurrence in baits relative to colony abundance in the environment. This was calculated as the number of baits occupied per site, divided by the number of pitfall traps occupied (sampling methods described above). To account for site‐level differences in sampling effort, we extracted estimated marginal mean ecological dominance scores for each ant species from a LMER that included sampling effort as a covariate and site as a random effect (additional details below and in Appendix S1).

#### 2.2.4 Discovery Ability

Resource discovery ability, or the ability to locate food quickly by foraging efficiently or occurring in high abundance, was also evaluated with baiting. Discovery ability was calculated as the time at which each taxon was first observed in each bait (Gibb and Hochuli 2004; Stuble et al. 2013). Ants were considered to have greater food discovery ability when they arrived faster. Although baits were observed at different frequencies and for different lengths of time across sites, we sampled all baits within a site at the same time and therefore should not have biased the results towards any particular taxa, after accounting for site‐level differences in sampling effort. We also likely missed the exact time when ants discovered baits that were left out for extended periods of time, artificially constraining discovery times. For instance, as we did not observe baits overnight or more than once per day after the first 24 h, ants reported as discovering a bait at 48 h could have arrived any time between the 24‐ and 48‐h observations. Nonetheless, the relative order of bait discovery by different taxa should have been unaffected by such gaps in observations.

#### 2.2.5 Ant Traits Underlying Dominance and Discovery Ability

We quantified multiple traits hypothesized to underlie dominance and discovery ability. We measured worker body size as the maximum head width in full face view (to the nearest 0.1 mm) of 12–327 workers of each taxon collected in pitfall traps, as well as the fresh body mass of 5–21 (16.2 ± 7.0 SD) workers of each taxon collected from the field. We calculated recruitment rate as the mean number of ants per observation at each bait (based on bait sampling described above) and worker biomass as mean ant body mass multiplied by recruitment rate. Ants could thus have high worker biomass from a large body size, high recruitment rate, or both.

#### 2.2.6 Meta‐Analysis

To compare the results from this study with past studies, we reviewed and quantitatively summarized the literature on dominance‐discovery trade‐offs in ant communities. On March 5, 2025, we identified studies to include by searching ISI *Web of Science* main library with the “All fields” search terms “dominan* AND discover* AND (‘trade‐off’ OR ‘tradeoff’) AND ‘ant’ AND compet*”. We cross‐referenced this search with articles cited in two previous papers that had compiled data on dominance and discovery abilities across studies or locations (Parr and Gibb 2012; Sheard et al. 2020). The search generated 200 unique papers. To be included in the meta‐analysis, the paper had to be published in English in a peer‐reviewed journal and focused on interspecific competition among ants. We eliminated 93 studies that did not meet these criteria. The remaining 107 studies were further assessed and excluded if: (i) one or more forms of dominance (behavioral, numerical, or ecological) or discovery ability were not measured, (ii) fewer than four unique ant taxa were compared (rendering calculations of correlation coefficients unreliable), or (iii) competition for food was not measured (e.g., competition was instead measured for other resources such as nesting space). After exclusion, 21 studies with extractable data remained. These data sources are listed in Table 1 and Appendix S1: Table S9.

We calculated effect sizes from each study as Spearman's rank correlations between dominance and discovery ability. In cases where Spearman's coefficients were not reported, we extracted raw data from figures and tables to calculate Spearman's rank. While we would have preferred to use Pearson's correlations rather than Spearman's, as they are sensitive not only to relative ranks but also to the magnitude of differences between dominance and discovery scores, fewer than half of studies included information required to compute them.

When multiple effects were reported in the same study, we included all correlation coefficients separately if they were based on different forms of dominance (e.g., behavioral versus numerical), ant communities, or habitats. However, because only our empirical study presents correlations between individual‐level behavioral dominance and discovery ability, all correlations with behavioral dominance included in the meta‐analysis are based on the colony level. When discovery ability was calculated in multiple ways within the same ant community, we computed Spearman's rank from just one metric (the one that was most used across studies to make results as comparable as possible). When dominance and discovery were measured under different mediating conditions in one study (in the presence versus absence of parasitoids; Lebrun and Feener 2007), effect sizes were calculated under only one condition (for non‐host species).

### 2.3 Statistical Analysis

#### 2.3.1 Empirical Study

We tested for differences among taxa in dominance metrics, discovery ability, and associated traits (response variables) using Fisher's exact tests (for behavioral dominance) and linear mixed effects models (LMERs; for all other metrics). Each LMER included ant taxon as a fixed effect, sampling effort (total number of hours baits or pitfall traps were deployed) as a covariate, and site as a random effect, when relevant. These analyses and results are described in more detail in Appendix S1, as the exact differences among ants in each of these variables is not the focus of this study.

To test for correlations between dominance metrics and discovery ability, we computed Pearson's correlation coefficients, as they are sensitive to the magnitude of differences between dominance and discovery scores, rather than just their relative ranks. Correlations were based on Colley scores (for individual‐level behavioral dominance) or estimated marginal mean values obtained from the LMERs testing for differences among taxa (for all other metrics; Appendix S1: Section S2). We first tested for correlations between all forms of dominance, including individual‐ and colony‐level behavioral dominance, numerical dominance measured in baits and pitfall traps, and ecological dominance. We then tested for trade‐offs between each form of dominance and discovery ability.

Because there was a trade‐off between behavioral dominance and discovery ability (see Section 3), we tested whether the trade‐off was correlated with the hypothesized associated traits. We first used principal components analysis (PCA) to create a linear combination of individual‐level behavioral dominance and discovery ability. While colony‐level behavioral dominance was negatively associated with discovery ability, it was strongly correlated with individual‐level dominance (see Section 3). We therefore just included one behavioral dominance metric (individual‐level) for the PCA. The first principal component (PC1) explained 96.7% of the covariation, being positively correlated with behavioral dominance (*r* = 0.98, *t* = 9.32, *p* = 0.003) and negatively correlated with discovery ability (*r* = −0.98, *t* = −9.32, *p* = 0.003; Appendix S1: Figure S6). Using PC1 as a proxy for the trade‐off, we computed Pearson's correlation coefficients between PC1 and each measured trait.

Statistical analyses were conducted in R v 4.0.2 (R Core Team 2024). Colley scores were calculated using the ‘rate_colley()’ function in the ‘comperank’ package (Chasnovski 2020), and to conduct Fisher's exact tests, we used the ‘fisher. test()’ function (R Core Team 2024). To construct LMERs we used the ‘lmer()’ function in the ‘lme4’ package (Bates et al. 2015). We checked for normality of residuals by visually inspecting residual distributions and using the ‘shapiro.test()’ function (R Core Team 2024). To evaluate the significance of fixed effects, we conducted *F* tests using the ‘Anova()’ function in the ‘car’ package (Fox and Weisberg 2019). We then used the ‘emmeans()’ function in the ‘emmeans’ package to obtain estimated marginal means (Lenth 2021). We computed Pearson correlation coefficients and tested whether they were significantly different from zero using the ‘cor.test()’ function (R Core Team 2024). The PCA was done using the ‘prcomp()’ function in the ‘stats’ package (R Core Team 2024).

#### 2.3.2 Meta‐Analysis

We constructed meta‐analytical models using the ‘rma.mv()’ function in the ‘metafor’ package (Viechtbauer 2010). Fisher's *Z*‐transformation was first applied to Spearman's rank correlations to stabilize the variance among coefficients. These transformed values were the effect sizes in all models. To estimate the overall relationship between dominance and the discovery ability of food, we first built a meta‐analytical model with study identity as a random factor, and we then extracted the predicted mean value. Next, to test whether the relationship between dominance and food discovery ability depended on dominance type (behavioral, numerical, or ecological), we constructed a meta‐analytical model with dominance type as a moderator. Because the number of ant species included in each study could influence the strength of the correlation, ant species number was also included as a moderator, and study identity was a random factor. We ran this model excluding the intercept and used the ‘summary()’ function to obtain predicted mean effect sizes for each moderator level (R Core Team 2024). Effect sizes were considered significantly different from zero when the 95% confidence intervals did not overlap with zero.

To test for publication bias, we conducted a modified Egger's regression (Egger et al. 1997) using the ‘lm()’ function, in which the residuals from our meta‐analytic model were the response and the standard error of the effect size was the predictor variable. Evidence for publication bias is present when the model intercept differs from zero. To estimate effect size heterogeneity, we calculated the *I* ^*2*^ statistic using the ‘i2_ml()’ function in the ‘orchaRd’ package (Nakagawa et al. 2023).

## 3 Results

### 3.1 Are Behavioral, Numerical, and Ecological Dominance Correlated?

**FIGURE 2:**
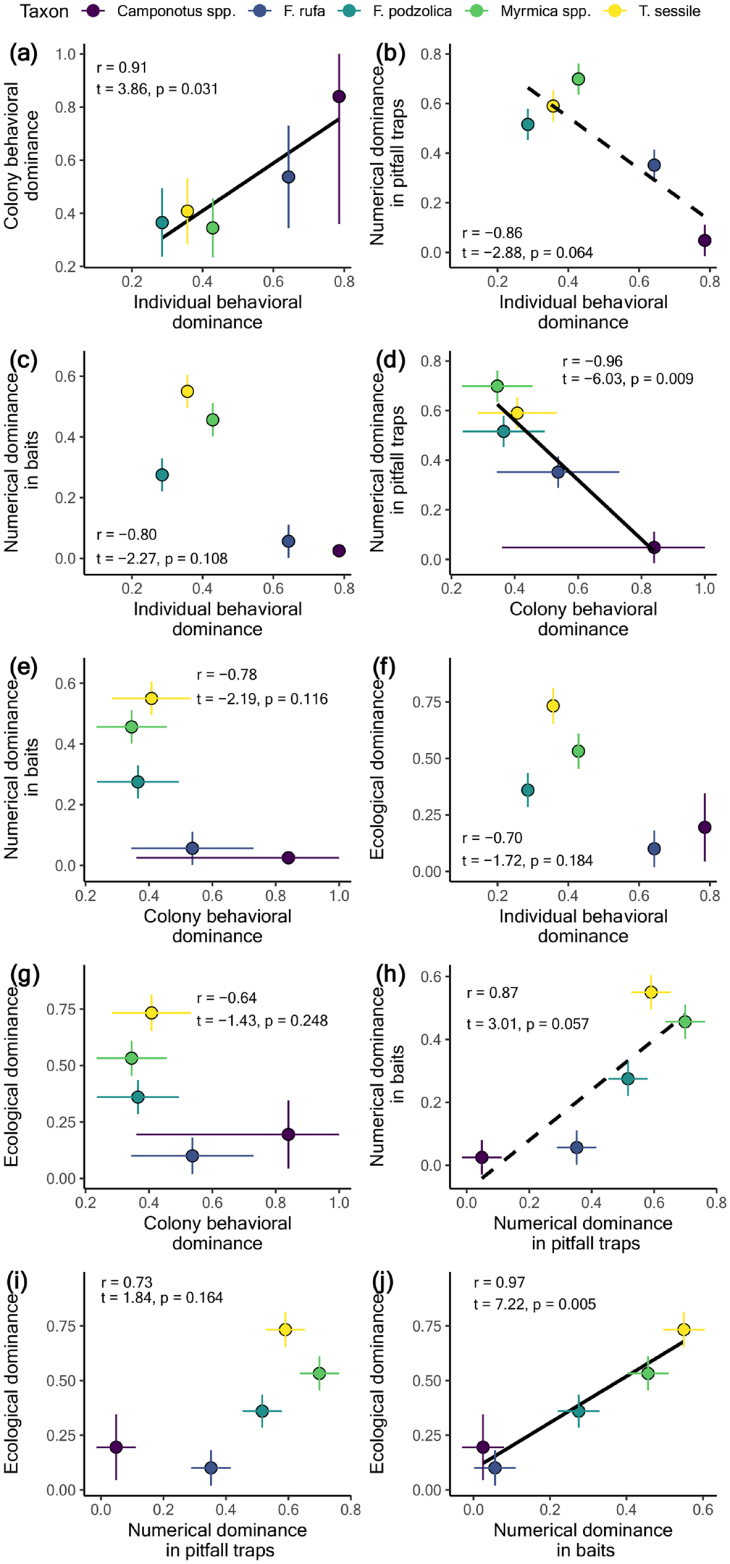
The relationship between (a) colony‐level behavioral and individual behavioral dominance, (b) numerical dominance in pitfall traps and individual behavioral dominance, (c) numerical dominance in baits and individual behavioral dominance, (d) numerical dominance in pitfall traps and colony‐level behavioral dominance, (e) numerical dominance in baits and colony‐level behavioral dominance, (f) ecological dominance and individual behavioral dominance, (g) ecological dominance and colony‐level behavioral dominance, (h) numerical dominance in baits and numerical dominance in pitfall traps, (i) ecological dominance and numerical dominance in pitfall traps, and (j) ecological dominance and numerical dominance in baits. Values are estimated marginal means±1SE.

Ant taxa differed in most dominance scores, which were correlated with each other in various ways. Taxa significantly differed in individual‐level behavioral dominance (Fisher's test: *p* < 0.001), numerical dominance in baits (LMER: *F*_4,60_ = 18.49, *p* < 0.001) and in pitfall traps (LMER: *F*_4,60_ = 19.78, *p* < 0.001), and ecological dominance (LMER: *F*_4,46_ = 10.19, *p* < 0.001), but not colony‐level behavioral dominance (LMER: *F*_4,117_ = 0.543, *p* = 0.705; Appendix S1: Section S2, Tables S2–S5, and Figures S2 and S3). Individual‐level behavioral dominance (from one‐on‐one assays) was positively correlated with colony‐level behavioral dominance (from baits; *r* = 0.91, *t* = 3.86, *p* = 0.031) but marginally negatively correlated with numerical dominance in pitfall traps (*r* = −0.86, *t* = −2.88, *p* = 0.064). Colony‐level behavioral dominance was negatively correlated with numerical dominance in pitfall traps (*r* = −0.96, *t* = −6.03, *p* = 0.009). Numerical dominance in baits was positively correlated with ecological dominance (*r* = 0.97, *t* = 7.22, *p* = 0.005) and marginally correlated with numerical dominance in pitfall traps (*r* = 0.87, *t* = 3.01, *p* = 0.057). No other correlations between dominance metrics were detected (Figure 2; Appendix S1: Table S6).

### 3.2 Do Any Forms of Dominance Trade Off With Discovery Ability?

**FIGURE 3:**
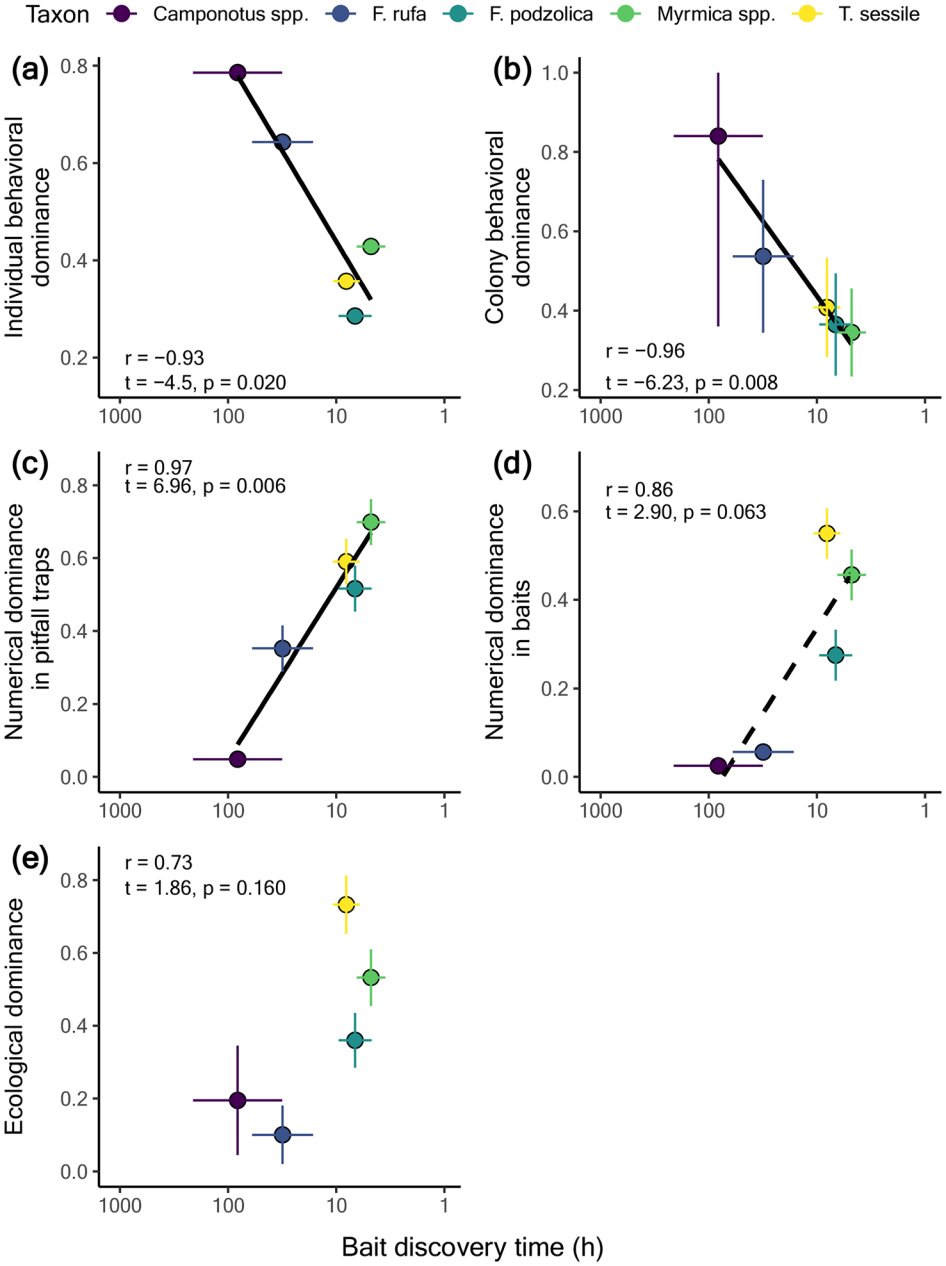
The relationship between discovery ability and (a) individual‐level behavioral dominance, (b) colony‐level behavioral dominance, (c) numerical dominance in baits, (d) numerical dominance in pitfall traps, and (e) ecological dominance. Values are estimated marginal means ±1SE.

Ants significantly differed in food discovery times (LMER: *F*_4,174_ = 4.51, *p* = 0.002; Appendix S1: Table S7, Figure S4), and there was a negative correlation between discovery ability and both individual‐level (*r* = −0.93, *t* = −4.5, *p* = 0.020) and colony‐level behavioral dominance (*r* = −0.96, *t* = −6.23, *p* = 0.008; Figure 3). In contrast, discovery ability was positively correlated with numerical dominance in pitfall traps (*r* = 0.97, *t* = 6.96, *p* = 0.006) and marginally correlated with numerical dominance in baits (*r* = 0.86, *t* = 2.90, *p* = 0.063; Figure 3). Discovery ability was uncorrelated with ecological dominance (*r* = 0.73, *t* = 1.86, *p* = 0.160; Figure 3).

### 3.3 What Are the Traits Associated With the Dominance‐Discovery Trade‐Off?

**FIGURE 4:**
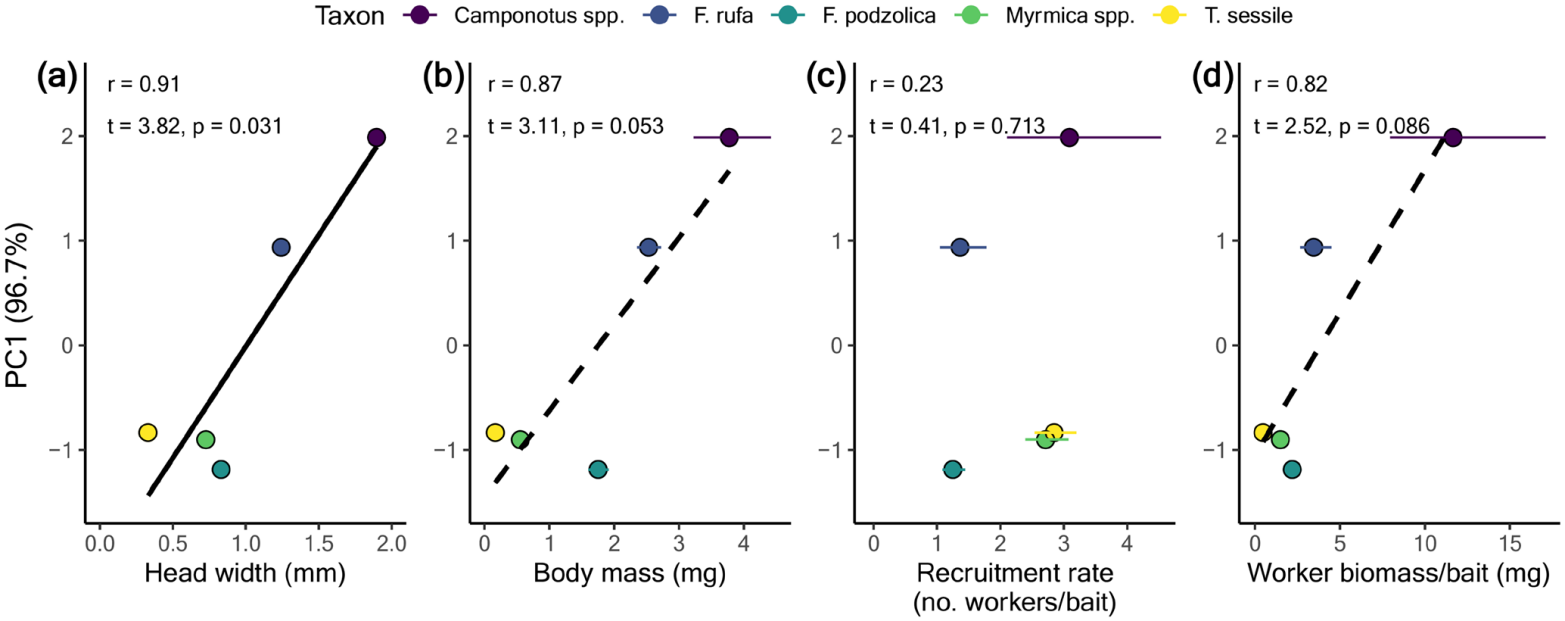
The relationship between PC1, which explains 96% of the variation in individual‐level behavioral dominance and discovery ability, and (a) mean worker head width (mm), (b) mean worker body mass (mg), (c) recruitment rate (mean number of workers recruited to the baits), and (d) mean biomass of workers recruited to baits (mg). Values are estimated marginal means ±1SE.

Ants differed in all measured traits, and the dominance‐discovery trade‐off was associated with several of them. Specifically, taxa differed in worker head size (LM: *F*_1,967_ = 937.44, *p* < 0.001), body mass (LM: *F*_4,76_ = 186.39, *p* < 0.001), recruitment rate (LMER: *F*_4,185_ = 10.69, *p* < 0.001), and biomass of recruits (LMER: *F*_4,185_ = 55.11, *p* < 0.001) (Appendix S1: Table S8, Figure S5). PC1 was positively associated with worker head width (*r* = 0.91, *t* = 3.82, *p* = 0.031), worker body mass (*r* = 0.87, *t* = 3.11, *p* = 0.053), and biomass of recruits (*r* = 0.82, *t* = 2.52, *p* = 0.086), such that ants that were more behaviorally dominant but slower to discover resources tended to have larger heads and heavier bodies, and recruit a greater biomass of workers (Figure 4). However, there was no association between PC1 and recruit number (*r* = 0.23, *t* = 0.41, *p* = 0.713; Figure 4).

### 3.4 Meta‐Analysis

**FIGURE 5:**
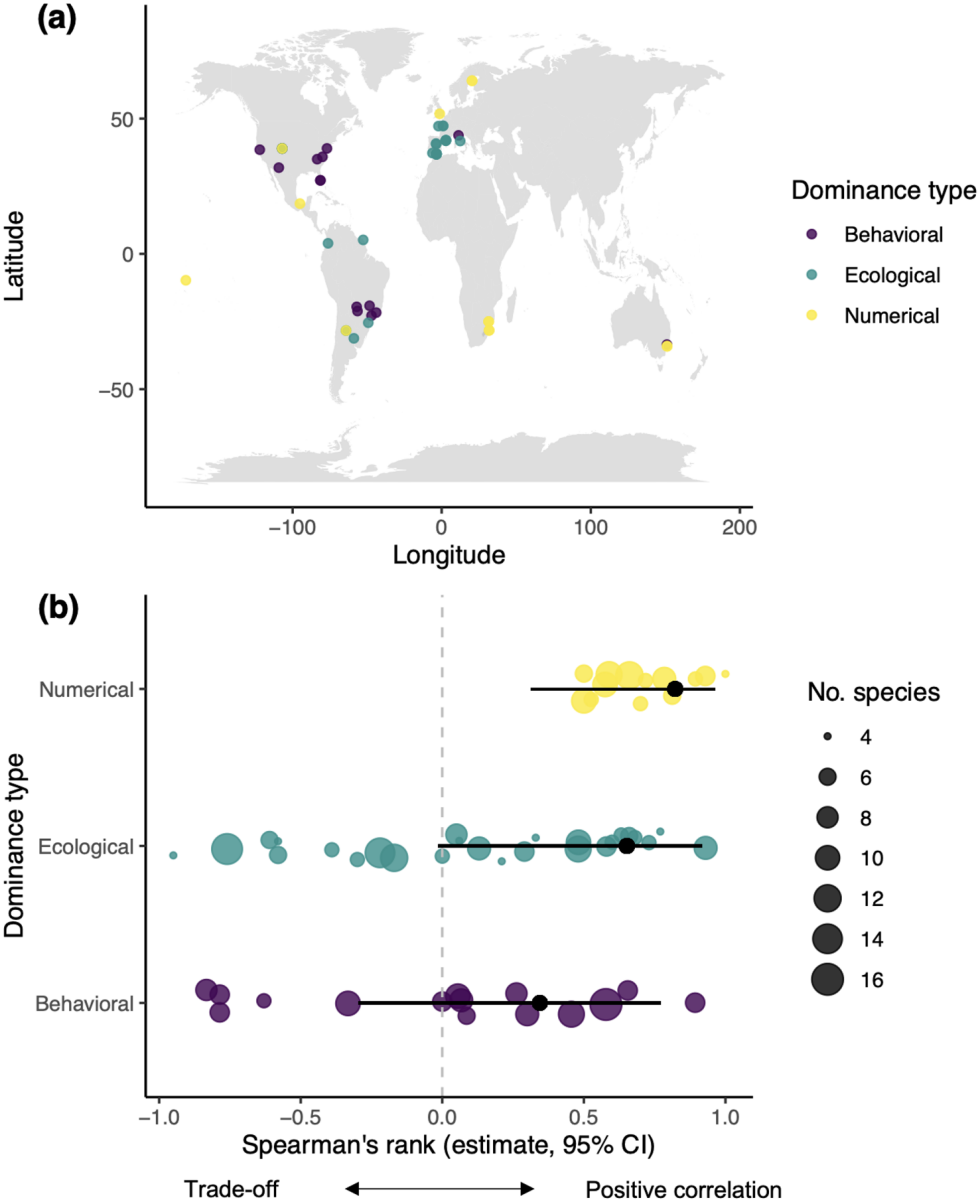
(a) World map of the studies included in the meta‐analysis and (b) the relationship between dominance type and Spearman's rank coefficients (back‐transformed weighted mean estimate, 95% CI) for the correlation between dominance and discovery ability. Point size indicates sample size (i.e., number of species used to obtain each correlation coefficient).

We identified 21 studies reporting on dominance and food discovery ability across ant species, quantifying 54 separate responses spanning more than 98° in latitude (Figure 5a; Appendix S1: Table S9). This is more than double the 23 responses identified in the prior meta‐analysis of dominance‐discovery trade‐offs among ants (Parr and Gibb 2012). Of all responses, 28% (15) were based on behavioral dominance, 24% (13) on numerical dominance, and 48% (26) on ecological dominance (Appendix S1: Table S9). The overall correlation between dominance and discovery ability did not significantly differ from zero, with a weighted mean Spearman's rank coefficient of +0.22 (95% CI: −0.10, +0.50). The strength of the correlation depended on dominance type (*Q* = 8.21, df = 3, *p* = 0.042) but not the number of species included in the study (*Q* = 2.40, df = 1, *p* = 0.121) (Figure 5b). Discovery ability was positively correlated with numerical dominance, with a predicted Spearman's rank correlation of +0.82 (95% CI: +0.31, +0.96) (Figure 5b). However, discovery ability was not significantly correlated with ecological or behavioral dominance, which had predicted Spearman's rank coefficients (respectively) of +0.65 (95% CI: −0.01, +0.92) and + 0.34 (95% CI: −0.30, +0.77) (Figure 5b).

We did not find evidence for publication bias in either meta‐analytical model. In both cases, the intercepts of the Egger's regressions did not significantly differ from zero (overall model: estimate = −0.17, *t* = −0.45, *p* = 0.654; model with dominance type and species number moderators: estimate = 0.10, *t* = 0.27, *p* = 0.785). However, we detected substantial heterogeneity among studies (overall model: *I* ^*2*^ = 65.40; model with dominance type and species number moderators: *I* ^*2*^ = 64.85).

## 4 Discussion

Ant taxa within this high‐elevation community exhibited a trade‐off between the ability to discover food quickly and both individual‐ and colony‐level behavioral dominance. Yet, different dominance metrics were not equivalent: while individual‐ and colony‐level behavioral dominance were positively correlated, as were ecological and numerical dominance (in baits), colony‐level behavioral dominance was negatively associated with numerical dominance (in pitfall traps). Instead of trading off with numerical or ecological dominance, discovery ability was positively associated with numerical dominance (in pitfall traps) and uncorrelated with ecological dominance. Whereas behaviorally dominant ants had larger body sizes and recruited a greater biomass of workers to baits (as a result of their larger body size), faster discoverers were more abundant (i.e., numerically dominant). Complementing these results, the meta‐analysis demonstrated that numerical dominance is significantly positively, rather than negatively, correlated with food discovery ability. Although the correlations of behavioral and ecological dominance with discovery ability were highly variable, they did not significantly differ from zero. Overall, these findings demonstrate that ants can be dominant in different ways, explaining in part why past studies may have found inconsistent evidence for dominance‐discovery trade‐offs (Parr and Gibb 2012; Stuble et al. 2017; Sheard et al. 2020). Yet, the dominance‐discovery trade‐off is still far from the rule, and many additional mechanisms likely play an equal, if not stronger, role in structuring ant communities.

The trade‐off between behavioral dominance and the ability to discover food in the focal ant community aligns with findings from some past studies (Fellers 1987; Savolainen and Vepsäläinen 1988; Parr and Gibb 2010). The trade‐off occurred with both individual‐ and colony‐level behavioral dominance, resulting in the ants falling into two groups: behavioral dominants (*Camponotus* spp. and *F. rufa* ), which are highly aggressive at baits but slow to discover food, and behavioral subordinates (*Myrmica* spp., *F. podzolica* , and *T. sessile* ), which excel at discovering food quickly and forage more opportunistically. We acknowledge, however, that infrequent interactions between certain pairs of taxa (especially taxa with low numerical dominance) limited the precision of behavioral dominance estimates. Moreover, because colony‐level dominance was based on sequential replacements of ants at baits, rather than direct observations of ant behavior, a portion of these replacements could have resulted from random abandonment or been influenced by differences in worker recruitment rates, as ants tend to be more aggressive in groups (Wenseleers et al. 2002; Carpintero and Reyes‐López 2008). Nonetheless, the strong negative correlation between discovery ability and both individual‐ and colony‐level dominance provides compelling support for a trade‐off. By allowing ants to excel in different aspects of resource acquisition (i.e., interference versus exploitation competition), the behavioral dominance‐discovery trade‐off may play a key role in structuring some ant communities such as this one. Yet, our review of the literature demonstrates that although the trade‐off occasionally occurs (11.1% of responses), most ant communities show no significant correlation between dominance and discovery ability (76.9%), or even a positive one (13.0%).

Importantly, numerical dominance was positively, rather than negatively, associated with discovery ability in both the empirical study and meta‐analysis. Abundant ants are likely better able to discover food simply by chance, and numerical dominance indeed serves as a predictor of discovery ability in some ant communities (e.g., Lebrun and Feener 2007; Pearce‐Duvet et al. 2011; Segev and Ziv 2012; Stuble et al. 2017). Yet, many studies still test for dominance‐discovery trade‐offs based on numerical dominance, likely causing us to underestimate the frequency of these trade‐offs across ant communities (e.g., Parr and Gibb 2012; Sheard et al. 2020) (Figure 5b; Appendix S1: Table S9). Our findings suggest that a better approach is to focus on behavioral dominance when testing for discovery trade‐offs, and that the trade‐off could even be reframed as one between behavioral and numerical dominance.

Although past studies have hypothesized that ecological dominance depends on a combination of both behavioral and numerical dominance, ecological dominance was only correlated with numerical dominance (measured in baits) in our empirical study. This is consistent with another recent finding that ecological dominance depends more on ant abundance and activity (i.e., numerical dominance) than on behavioral dominance (Stuble et al. 2017). We note, however, that numerical and ecological dominance may sometimes be spuriously correlated, as ecological dominance is often calculated based on the numerical dominance in baits divided by the numerical dominance in pitfall traps. Ecological dominance is known to be associated with the presence of multiple queens per colony, higher tolerance of temperature and desiccation risk, and faster walking speeds, but not with worker body size or dimorphism (Boulay et al. 2014; Tschá and Pie 2019). As some of these traits may also be linked to numerical and behavioral dominance, but others may not, alternate relationships between the three dominance metrics could occur in other communities.

Better understanding the traits underlying dominance and discovery ability is needed to determine the factors structuring ant communities. We found that behaviorally dominant ants have larger body sizes and recruit a greater worker biomass to baits (because of having larger body sizes), explaining how they excel in interference competition. Previous studies have similarly found associations between behavioral dominance and large worker body size, as well as large colony size, the presence of multiple nests per colony, worker polymorphism, and collective foraging strategies (Palmer 2004; Retana et al. 2015; Jelley and Moreau 2023). We found that fast discoverers, in contrast, were numerically dominant, with a greater number of colonies in the environment, resulting in increased worker density. This finding aligns with previous predictions (Lebrun and Feener 2007). A dominance‐discovery trade‐off could thus have emerged because the traits associated with behavioral dominance (e.g., body size) influence ant foraging distance and speed. Larger ants generally forage farther distances, but ants with smaller body sizes and smaller legs relative to body size can forage faster in complex habitats (“size grain hypothesis”; Kaspari and Weiser 1999; Farji‐Brener et al. 2004). Yet, other unmeasured traits could also influence behavioral dominance and discovery ability (e.g., colony size, number of nests per colony, and foraging distance), demonstrating the importance of further investigating the traits underlying dominance and food discovery ability across a broader range of taxa. Identifying such traits can also help to predict when alternative relationships between dominance and discovery ability (e.g., the discovery‐defense strategy; Camarota et al. 2018; Antoniazzi et al. 2021) are likely to occur.

Several methodological constraints in both the empirical study and meta‐analysis may have limited our ability to detect correlations between some variables. In the empirical study, measurements of dominance were potentially influenced by small sample sizes, as certain taxa (e.g., *Camponotus* spp. and *F. rufa* group) occurred infrequently in baits. Combining species into a relatively small number of taxonomic groups (five), while allowing us to account for phylogenetic non‐independence, could have also reduced statistical power and limited our ability to detect significant correlations between traits. Nonetheless, the significant correlations between some variables (e.g., discovery ability and behavioral dominances) attests to how closely linked these traits are within this ant community. The choice to base analyses on Pearson's rather than Spearman's correlation coefficients could have also influenced the results, and although most studies in the meta‐analysis only reported Spearman's correlations, we believe that Pearson's correlations are more appropriate for testing for dominance‐discovery trade‐offs because they are not solely based on rankings. In the meta‐analysis, although we were able to broadly test for the effect of dominance type (behavioral, numerical, and ecological) on the strength of the association with discovery ability, there was still much variability among studies in how dominance and discovery scores were calculated (Table 1). This, as well as other potential methodological discrepancies among studies, may have limited our ability to detect dominance‐discovery trade‐offs across systems. There was indeed substantial heterogeneity across studies, suggesting that additional work is needed to further explain sources of variation in the strength of the correlation between dominance and discovery ability. Yet, our work demonstrates that dominance type is one source of variation in effect size across studies.

In summary, we have shown that behavioral, numerical, and ecological dominance are not interchangeable, suggesting that ants can be dominant in different ways. This raises the question: what does it mean to be dominant? Because numerical dominance is consistently positively, rather than negatively, associated with food discovery ability, future tests for dominance‐discovery trade‐offs should not be based on numerical dominance. Instead, behavioral dominance is a more appropriate metric to use when testing for the trade‐off. Yet, the meta‐analysis makes clear that even though a behavioral dominance‐discovery trade‐off occurs in some ant communities (11.1%), it is uncommon, at least at the coarse assemblage level. Because ants partition food resources as a function of biotic and abiotic context (e.g., enemy presence, resource size, time of day, and temperature; Cerdá et al. 1997, 1998; Lebrun and Feener 2007; Houadria et al. 2016), dominance‐discovery trade‐offs may be more important under certain conditions. Additional studies that examine how such factors mediate these dynamics will expand our understanding of when and where dominance‐discovery trade‐offs occur. For instance, studies conducted across larger spatial scales (e.g., elevational gradients) within the same region as our empirical study demonstrate that warmer sites have higher ant abundance and activity in pitfall traps, baits, and attending aphid colonies (Mooney et al. 2016; Nelson, Symanski, et al. 2019; Nelson, Pratt, et al. 2019). It is possible that climate alters ant dominance and discovery ability within these sites, although community composition does not change (Mooney et al. 2016; Nelson, Symanski, et al. 2019; Nelson, Pratt, et al. 2019).

Ultimately, studies that more clearly link differences in competition‐relevant traits to colony performance (e.g., colony survival, growth, and reproduction) and population dynamics are needed to delineate the mechanisms mediating ant coexistence and community composition. This will require measuring competition for other resources in addition to food, such as nesting space, which has previously received much less attention but is likely just as important. This study suggests, however, that the trade‐off between dominance and food discovery ability plays little role in structuring most ant communities.

## Author Contributions

**Annika S. Nelson:** conceptualization (equal), data curation (lead), formal analysis (lead), funding acquisition (lead), investigation (equal), methodology (equal), resources (equal), software (lead), visualization (lead), writing – original draft (lead), writing – review and editing (equal). **Kailen A. Mooney:** conceptualization (equal), formal analysis (supporting), funding acquisition (supporting), investigation (supporting), methodology (equal), resources (equal), writing – review and editing (equal).

## Conflicts of Interest

The authors declare no conflicts of interest.

## Supporting information


**Data S1:** Field site descriptions.
**Section S2**. Comparing dominance scores, discovery ability, and underlying traits.
**Table S1:** GPS coordinates, elevation, duration of pitfall trap sampling, duration of baiting, timing of observations of baits, taxa present in baits and/or pitfall traps, and taxa included in one‐on‐one assays in each site of the empirical study. Ant names are abbreviated as “*C*.spp.” for *Camponotus* spp., “*F.r*.” for *Formica rufa* group, “*M*.spp.” for *Myrmica* spp., “*T.s*.” for *Tapinoma sessile* , and “*F.p*.” for *Formica podzolica* .
**Table S2:** Matrix of outcomes from (a) staged one‐on‐one assays and (b) transitions among taxa at 20% honey baits. Ant names are abbreviated as “*C*.spp.” for *Camponotus* spp., “*F.r*.” for *Formica rufa* group, “*M*.spp.” for *Myrmica* spp., “*T.s*.” for *Tapinoma sessile* , and “*F.p*.” for *Formica podzolica* . Rows list the number of times each taxon won against all other taxa, and columns list the number of times each taxon lost. The greater number of wins for each taxon pair is bolded. The *p*‐values indicate whether the raw behavioral dominance scores (proportion of interactions won) and corrected scores (Colley scores for individual‐level dominance and estimated marginal means for colony‐level dominance) were significantly correlated.
**Table S3:** Results from multiple comparisons for differences in raw individual‐level behavioral dominance scores among ant taxa in staged one‐on‐one encounters, with *p‐*values from each Fisher's exact test corrected using the Bonferroni method. Note that because there was no significant difference among ants in raw colony‐level behavioral dominance scores, we did not conduct any follow‐up comparisons among individual pairs of taxa.
**Table S4:** Results from Tukey tests for multiple comparisons for differences in numerical dominance scores among ant taxa.
**Table S5:** Results from Tukey tests for multiple comparisons for differences in ecological dominance scores among ant taxa.
**Table S6:** Results from separate tests for correlations between dominance metrics.
**Table S7:** Results from Tukey tests for multiple comparisons for differences in discovery ability among ant taxa.
**Table S8:** Results from Tukey tests for multiple comparisons for differences in (a) forager head width, (b) forager body mass, (c) forager recruitment rate to baits, and (d) biomass of recruited foragers.
**Table S9:** Summary of studies measuring the correlation (*r*_*s*_) between dominance and discovery ability among ant species within an ecological community.
**Figure S1:** Diagram of the layout of bait and pitfall trap stations within each site. Baits were spaced 10 m apart along a 2 x 5 grid. Pitfall traps were deployed along the same grid but staggered 1 m diagonally to the right of each bait and deployed 24 h after baiting was completed, to avoid interference between the baiting and pitfall trapping.
**Figure S2:** Boxplots of the numerical dominance (y‐axis) of each ant taxon (x‐axis), measured as the proportion of (a) baits and (b) pitfall traps occupied per site.
**Figure S3:** Boxplots of ant ecological dominance, measured as the proportion of baits occupied out of all the stations where that taxon occurred in the pitfall traps (y‐axis), of each of the five taxa (x‐axis).
**Figure S4:** Boxplots of ant resource discovery ability, measured as the time (number of hours; y‐axis) at which each taxon (x‐axis) was first observed at each 20% honey bait where it occurred. Note that the y‐axis is reversed, such that faster resource discoverers (i.e., ants that took fewer hours to discover resources) are shown higher on the y‐axis.
**Figure S5:** Boxplots of ant traits hypothesized to underlie the dominance‐discovery trade‐off, including (a) forager head width (mm), (b) forager body mass (mg), (c) forager recruitment rate (mean number of ants per observation of a bait), and (d) the mean biomass of recruited foragers (mg; calculated as body mass x forager recruitment rate).
**Figure S6:** The relationship between PC1 and (a) individual‐level behavioral dominance and (b) discovery ability. PC1 explained 96.7% of the covariation in behavioral dominance and discovery ability.

## Data Availability

All data, metadata, and R scripts are available in Dryad Digital Repository and can be accessed using the following link: https://doi.org/10.5061/dryad.c866t1ggn.
