# Supplementary material for: Different Aspects of Dominance Are Not Equivalent When Testing for Trade‐Offs in Ant Communities
Source: Ecol Evol. 2025 Sep 21;15(9):e72207. doi: 10.1002/ece3.72207 (PMC12450609; doi:10.1002/ece3.72207)
Supplement: Supplementary file 1 — Data S1: Field site descriptions. Section S2. Comparing dominance scores, discovery ability, and underlying traits. Table S1: GPS coordinates, elevation, duration of pitfall trap sampling, duration of baiting, timing of observations of baits, taxa present in baits and/or pitfall traps, and taxa included in one‐on‐one assays in each site of the empirical study. Ant names are abbreviated as “C.spp.” for Camponotus spp., “F.r.” for Formica rufa group, “M.spp.” for Myrmica spp., “T.s.” for Tapinoma sessile , and “F.p.” for Formica podzolica . Table S2: Matrix of outcomes from (a) staged one‐on‐one assays and (b) transitions among taxa at 20% honey baits. Ant names are abbreviated as “C.spp.” for Camponotus spp., “F.r.” for Formica rufa group, “M.spp.” for Myrmica spp., “T.s.” for Tapinoma sessile , and “F.p.” for Formica podzolica . Rows list the number of times each taxon won against all other taxa, and columns list the number of times each taxon lost. The greater number of wins for each taxon pair is bolded. The p‐values indicate whether the raw behavioral dominance scores (proportion of interactions won) and corrected scores (Colley scores for individual‐level dominance and estimated marginal means for colony‐level dominance) were significantly correlated. Table S3: Results from multiple comparisons for differences in raw individual‐level behavioral dominance scores among ant taxa in staged one‐on‐one encounters, with p‐values from each Fisher's exact test corrected using the Bonferroni method. Note that because there was no significant difference among ants in raw colony‐level behavioral dominance scores, we did not conduct any follow‐up comparisons among individual pairs of taxa. Table S4: Results from Tukey tests for multiple comparisons for differences in numerical dominance scores among ant taxa. Table S5: Results from Tukey tests for multiple comparisons for differences in ecological dominance scores among ant taxa. Table S6: Results from separate tests for cor [file ECE3-15-e72207-s001.docx]

**Appendix S1**

**Section S1. Field site descriptions**

**Table S1.** GPS coordinates, elevation, duration of pitfall trap sampling, duration of baiting, timing of observations of baits, taxa present in baits and/or pitfall traps, and taxa included in one-on-one assays in each site of the empirical study. Ant names are abbreviated as “*C.*spp.” for *Camponotus* spp., “*F.r.*” for *Formica rufa* group, “*M.*spp.” for *Myrmica* spp., “*T.s.*” for *Tapinoma sessile*, and “*F.p.*” for *Formica podzolica*.

| Study site | Latitude | Longitude | Elevation (m) | Pitfall trap duration (h) | Bait duration (h) | Observation timing | Taxa present in baits and/or pitfall traps | Taxa in one-on-one assays |
| --- | --- | --- | --- | --- | --- | --- | --- | --- |
| AJF | 38.96002 | -106.98078 | 2,995 | 56 | 3 | AM | *F.r.*, *M.*spp., *T.s.*, *F.p.* | None |
| GRD1 | 38.97343 | -106.99633 | 2,929 | 44 | 49 | AM, PM | *F.r.*, *M.*spp., *T.s.*, *F.p.* | None |
| GRD2 | 38.96391 | -106.99313 | 2,922 | 53 | 98 | AM, PM | *F.r.*, *M.*spp., *T.s.*, *F.p.* | *F.r.*, *M.*spp., *T.s.*, *F.p.* |
| GRD3 | 38.96398 | -106.99376 | 2,919 | 53 | 50 | AM | *C.*spp., *F.r.*, *M.*spp., *T.s.*, *F.p.* | *M.*spp., *T.s.*, *F.p.* |
| JFRM1 | 38.96069 | -106.98428 | 2,965 | 51 | 96 | AM, PM | C.spp., *F.r.*, *M.*spp., *T.s.*, *F.p.* | *C.*spp., *F.r.*, *M.*spp., *T.s.*, *F.p.* |
| JFRM2 | 38.96118 | -106.98493 | 2,974 | 49 | 48 | AM | *F.r.*, *M.*spp., *T.s.*, *F.p.* | None |
| JFRM3 | 38.96228 | -106.98641 | 2,984 | 49 | 121 | AM | *C.*spp., *F.r.*, *M.*spp., *T.s.*, *F.p.* | None |
| JFRM4 | 38.95993 | -106.98274 | 2,967 | 48 | 96 | AM | *F.r.*, *M.*spp., *T.s.*, *F.p.* | *F.r.*, *M.*spp., *T.s.*, *F.p.* |
| JFTR | 38.95964 | -106.98405 | 2,945 | 51 | 98 | AM, PM | *C.*spp., *F.r.*, *M.*spp., *T.s.*, *F.p.* | *C.*spp., *F.r.*, *M.*spp., *T.s.*, *F.p.* |
| NJFTH | 38.96826 | -106.99477 | 2,927 | 72 | 27 | AM, PM | *C.*spp., *M.*spp., *T.s.*, *F.p.* | *C.*spp., *M.*spp., *T.s.* |
| SJFTH | 38.96538 | -106.99344 | 2,924 | 70 | 29 | AM, PM | *C.*spp., *F.r.*, *M.*spp., *T.s.*, *F.p.* | *C.*spp., *F.r.*, *T.s.*, *F.p.* |
| SNOD | 38.94108 | -106.98347 | 2,879 | 26 | 1.5 | AM | *F.r.*, *F.p.* | None |
| SNTH1 | 38.92643 | -106.96301 | 2,933 | 45 | 121 | AM | *F.r.*, *M.*spp., *T.s.*, *F.p.* | *F.r.*, *T.s.*, *F.p.* |
| SNTH2 | 38.92608 | -106.96255 | 2,917 | 45 | 72 | AM | *F.r.*, *M.*spp., *T.s.*, *F.p.* | *F.r.*, *T.s.*, *F.p.* |
| SNTH3 | 38.92490 | -106.96237 | 2,929 | 47 | 48 | AM | *F.r.*, *M.*spp., *T.s.*, *F.p.* | *F.r.*, *T.s.*, *F.p.* |
| SNTH4 | 38.92487 | -106.96262 | 2,932 | 47 | 48 | AM | *F.r.*, *M.*spp., *T.s.*, *F.p.* | *F.r.*, *T.s.*, *F.p.* |

**Fig. S1.** Diagram of the layout of bait and pitfall trap stations within each site. Baits were spaced 10 m apart along a 2 x 5 grid. Pitfall traps were deployed along the same grid but staggered 1 m diagonally to the right of each bait and deployed 24 h after baiting was completed, to avoid interference between the baiting and pitfall trapping.

**Section S2. Comparing dominance scores, discovery ability, and underlying traits**

Behavioral dominance

We tested for differences among ants in raw behavioral dominance scores, measured both at the individual (from staged one-on-one encounters) and colony levels (from transitions among ants at baits). To do so, we first constructed matrices summarizing the outcomes of interactions among all possible pairs of ant taxa, with winners of each interaction assigned a score of 1, losers assigned a score of 0, and ties included as ½ points for both taxa (Table S2). Raw behavioral dominance scores were calculated as the total number of wins and ties each taxon had against all other taxa, divided by the total number of interactions (Table S2). We then constructed Fisher’s exact tests to test for differences in behavioral dominance among ants using the ‘fisher.test()’ function in the ‘stats’ package (R Core Team 2020). Before performing the analysis, all counts of wins and losses were rounded to the nearest integer in cases where the inclusion of ties as ½ points resulted in non-integer values.

Ants significantly differed in raw individual-level behavioral dominance scores (Fisher’s test: *p* < 0.001). To determine which specific pairs of taxa differed, we conducted post-hoc tests with Bonferroni corrections using the ‘fisher.multcomp()’ function in the RVAideMemoire package (Hervé 2022). We found that although *Camponotus* spp. and *F. rufa* did not significantly differ in behavioral dominance, they were both significantly more dominant than the other three taxa (Table S3). There were no detectable differences in behavioral dominance between *F. podzolica*, *T. sessile*, and *Myrmica* spp. (Table S3). These findings demonstrate that the ant taxa to fall into two behavioral dominance categories: dominants (*Camponotus* spp. and *F. rufa*) and subordinates (*F. podzolica*, *T. sessile*, and *Myrmica* spp.). Because dominance scores can be biased, however, by differences in sample size among paired taxa, we corrected raw individual-level behavioral dominance scores using Colley’s bias-free method. Colley scores take into account not only the number of wins and losses of each ant but also the relative dominance scores of the two opponents (Lebrun and Feener 2007, Stuble et al. 2013) to estimate the overall individual-level behavioral dominance score. Individual-level Colley scores were positively correlated with raw individual-level behavioral dominance scores, suggesting our measurements of individual-level dominance are robust (r = 0.97, *t* = 6.73, *p* = 0.007).

In contrast to individual-level behavioral dominance, raw colony-level behavioral dominance scores showed no detectable difference among ant taxa (Fisher’s test: *p* = 0.410). However, this result is likely driven, at least in part, by the infrequent occurrence and number of interactions involving the two behaviorally dominant ants (*Camponotus* spp. and *F. rufa*) in the baits (Table S2). These dominance scores could be biased by not only differences in sample size among taxa but also repeated observations of interactions among ants within the same bait. To account for this, we calculated the proportion of interactions taxa won against each competitor within each bait. We then constructed a linear mixed effects model (LMER) using the ‘lmer()’ function in the ‘lme4’ package (Bates et al. 2015) to test whether the proportion of interactions won per bait differed across ant taxa, depended on the identity of the competitor, and the ant taxon x competitor identity interaction. Site and bait within site were included as random effects. We found that ant taxa did not significantly differ in colony-level behavioral dominance scores (LMER: *F*_4,117_ = 0.543, *p* = 0.705). There was also no detectable main effect of competitor identity (LMER: *F*_4,117_ = 0.543, *p* = 0.705) or an ant taxon x competitor identity interaction (LMER: *F*_5,159_ = 0.543, *p* = 0.447). We extracted estimated marginal mean colony-level dominance scores for each ant species, allowing us to estimate colony-level dominance while accounting for the other factors included in this model (Table S2). Colony-level estimated marginal mean scores were positively correlated with raw colony-level behavioral dominance scores (r = 0.99, *t* = 12.54, *p* = 0.001). As both the adjusted individual- and colony-level dominance scores are strongly positively correlated with raw values, we used corrected values for all analyses presented in the main text.

**Table S2.** Matrix of outcomes from (a) staged one-on-one assays and (b) transitions among taxa at 20% honey baits. Ant names are abbreviated as “*C.*spp.” for *Camponotus* spp., “*F.r.*” for *Formica rufa* group, “*M.*spp.” for *Myrmica* spp., “*T.s.*” for *Tapinoma sessile*, and “*F.p.*” for *Formica podzolica*. Rows list the number of times each taxon won against all other taxa, and columns list the number of times each taxon lost. The greater number of wins for each taxon pair is bolded. The *p*-values indicate whether the raw behavioral dominance scores (proportion of interactions won) and corrected scores (Colley scores for individual-level dominance and estimated marginal means for colony-level dominance) were significantly correlated.

| Winner | Loser | | | | | Total wins | Proportion won | Colley score |
| --- | --- | --- | --- | --- | --- | --- | --- | --- |
|  | *C.*spp. | *F.r.* | *M.*spp. | *T.s.* | *F.p.* |  |  |  |
| (a) Individual-level behavioral dominance in staged one-on-one encounters | | | | | | | | |
| *C.*spp. | - | **4.5** | **5** | **4** | **7** | 20.5 | 0.932 | 0.786 |
| *F.r.* | 1.5 | - | **6.5** | **17.5** | **11** | 36.5 | 0.830 | 0.643 |
| *M.*spp. | 0 | 1.5 | - | **4** | **5** | 10.5 | 0.375 | 0.429 |
| *T.s.* | 0 | 0.5 | 1 | - | **5** | 6.5 | 0.181 | 0.357 |
| *F.p.* | 0 | 1 | 5 | 4 | - | 10.0 | 0.263 | 0.286 |
| Total losses | 1.5 | 7.5 | 17.5 | 29.5 | 28 |  |  | *P* = 0.007 |
|  | | | | | | Total wins | Proportion won | Estimated marginal mean |
| (b) Colony-level behavioral dominance in baits | | | | | |  |  |  |
| *C.*spp. | - | 0 | **1** | 0 | 0 | 1 | 1.000 | 0.843 |
| *F.r.* | 0 | - | **2** | **2** | **2** | 6 | 0.667 | 0.573 |
| *M.*spp. | 0 | **2** | - | 23 | 10 | 35 | 0.437 | 0.343 |
| *T.s.* | 0 | 1 | **28** | - | **9** | 38 | 0.551 | 0.403 |
| *F.p.* | 0 | 0 | **14** | 6 | - | 20 | 0.488 | 0.339 |
| Total losses | 0 | 3 | 45 | 31 | 21 |  |  | *P* = 0.001 |

**Table S3.** Results from multiple comparisons for differences in raw individual-level behavioral dominance scores among ant taxa in staged one-on-one encounters, with *p-*values from each Fisher’s exact test corrected using the Bonferroni method. Note that because there was no significant difference among ants in raw colony-level behavioral dominance scores, we did not conduct any follow-up comparisons among individual pairs of taxa.

| Taxon pair | *p*-value |
| --- | --- |
| *Camponotus* spp. – *F. rufa* | 1.00 |
| *Camponotus* spp. – *Myrmica* spp. | 0.001 |
| *Camponotus* spp. – *F. podzolica* | <0.001 |
| *Camponotus* spp. – *T. sessile* | <0.001 |
| *F. rufa* – *Myrmica* spp. | 0.001 |
| *F. rufa* – *F. podzolica* | <0.001 |
| *F. rufa* – *T. sessile* | <0.001 |
| *Myrmica* spp. – *F. podzolica* | 1.00 |
| *Myrmica* spp. – *T. sessile* | 0.919 |
| *F. podzolica – T. sessile* | 1.00 |

Numerical dominance

We tested for differences among ants in numerical dominance in baits and pitfall traps (proportion of baits or pitfall traps occupied per site) with separate LMERs. Both models included ant taxon as a fixed effect, and to account for differences in sampling effort across sites, we included the number of hours that baits or pitfall traps were deployed as a covariate and site as a random effect. We found that ants significantly differed in numerical dominance in baits (LMER: *F*_4,60_ = 18.49, *p* < 0.001), and such effects depended on sampling effort (LMER: *F*_4,60_ = 7.30, *p* = 0.017). Ants also significantly differed in numerical dominance in pitfall traps (LMER: *F*_4,60_ = 19.78, *p* < 0.001; Fig. S2), regardless of sampling effort (LMER: *F*_1,14_ = 0.13, *p* = 0.725). We conducted Tukey post-hoc tests to test for differences between each pair of taxa using the ‘glht()’ function in the ‘multcomp’ package (Hothorn et al. 2008). From both the bait and pitfall trap data, we found that *Camponotus* spp. was significantly less numerically dominant than the other ant taxa, except for *F. rufa* in the baits (but not pitfall traps; Table S4; Fig. S2). *F. rufa* was significantly less numerically dominant than *F. podzolica* in the baits (but not pitfall traps) and less numerically dominant than *Myrmica* spp. and *T. sessile* in both the baits and pitfall traps (Table S4; Fig. S2). There were no detectable pairwise differences in numerical dominance between *Myrmica* spp., *T. sessile*, or *F. podzolica* in the pitfall traps, although *F. podzolica* was significantly less numerically dominant than *T. sessile* in the baits (Table S4; Fig. S2).

**Table S4.** Results from Tukey tests for multiple comparisons for differences in numerical dominance scores among ant taxa.

| Taxon pair | *z* score | *p*-value |
| --- | --- | --- |
| Numerical dominance in baits |  |  |
| *Camponotus* spp. – *F. rufa* | -0.41 | 0.994 |
| *Camponotus* spp. – *Myrmica* spp. | -5.61 | <0.001 |
| *Camponotus* spp. – *F. podzolica* | -3.25 | 0.010 |
| *Camponotus* spp. – *T. sessile* | -6.83 | <0.001 |
| *F. rufa* – *Myrmica* spp. | -5.20 | <0.001 |
| *F. rufa* – *F. podzolica* | -2.84 | 0.036 |
| *F. rufa* – *T. sessile* | -6.42 | <0.001 |
| *Myrmica* spp. – *F. podzolica* | 2.36 | 0.127 |
| *Myrmica* spp. – *T. sessile* | -1.22 | 0.740 |
| *F. podzolica – T. sessile* | -3.58 | 0.003 |
|  |  |  |
| Numerical dominance in pitfall traps |  |  |
| *Camponotus* spp. – *F. rufa* | -3.77 | 0.002 |
| *Camponotus* spp. – *Myrmica* spp. | -8.08 | <0.001 |
| *Camponotus* spp. – *F. podzolica* | -5.81 | <0.001 |
| *Camponotus* spp. – *T. sessile* | -6.73 | <0.001 |
| *F. rufa* – *Myrmica* spp. | -4.31 | <0.001 |
| *F. rufa* – *F. podzolica* | -0.16 | 0.248 |
| *F. rufa* – *T. sessile* | -0.24 | 0.026 |
| *Myrmica* spp. – *F. podzolica* | 0.18 | 0.155 |
| *Myrmica* spp. – *T. sessile* | 0.11 | 0.660 |
| *F. podzolica – T. sessile* | -0.07 | 0.889 |


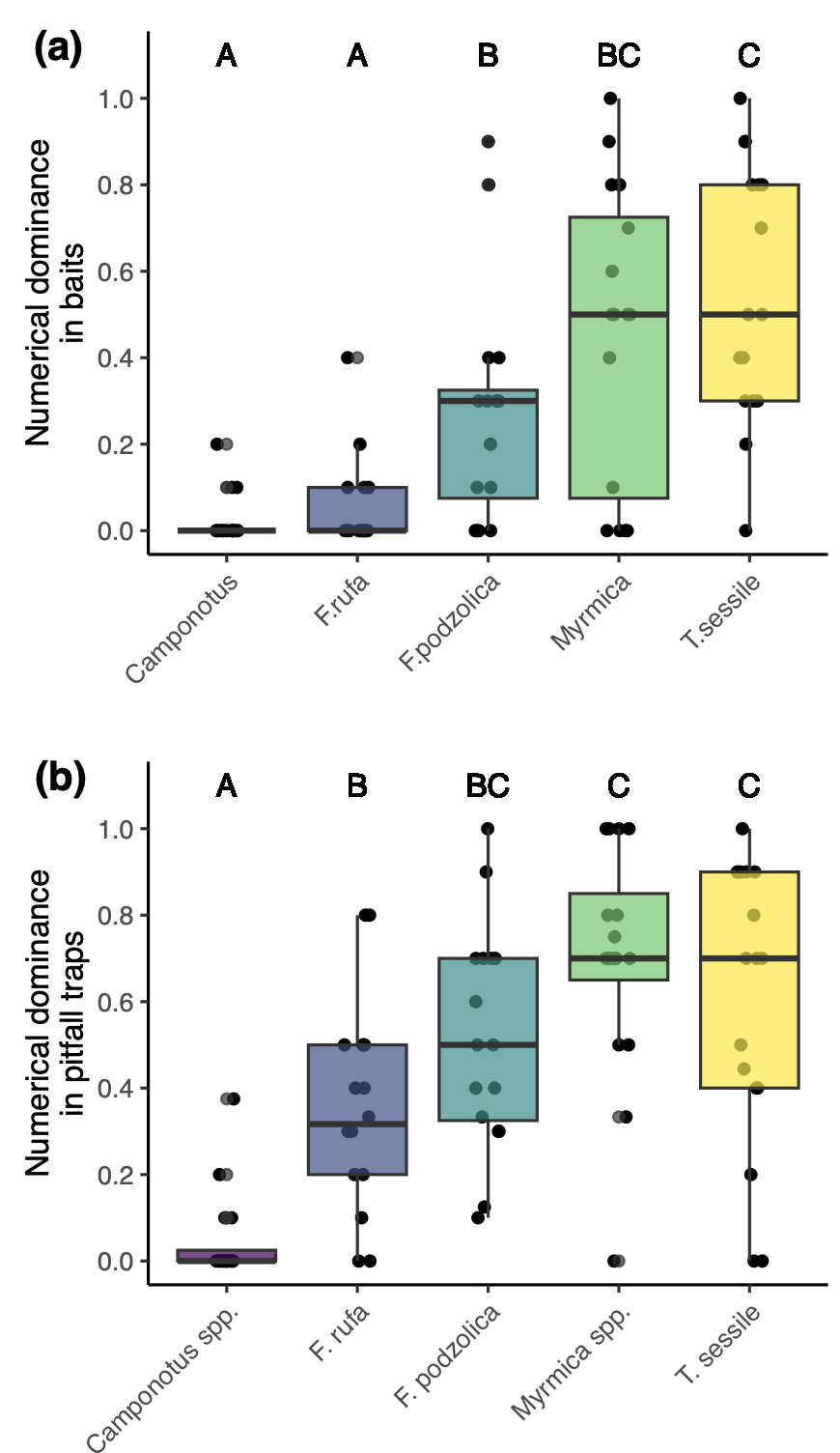


**Fig. S2.** Boxplots of the numerical dominance (y-axis) of each ant taxon (x-axis), measured as the proportion of (a) baits and (b) pitfall traps occupied per site.

Ecological dominance

We tested for differences among ants in ecological dominance (proportion of baits occupied out of all pitfall trap stations where that taxon occurred) with an LMER. The model included ant taxon as a fixed effect, and to account for differences in sampling effort across sites, we included the number of hours that baits were deployed as a covariate and site as a random effect. We found that ants significantly differed in ecological dominance (LMER: *F*_4,46_ = 10.19, *p* < 0.001; Fig. S3), regardless of sampling effort (LMER: *F*_1,14_ = 1.22, *p* = 0.287). To determine which ant taxa differed, we conducted Tukey post-hoc tests using the ‘glht()’ function in the ‘multcomp’ package (Hothorn et al. 2008). Although *Camponotus* spp. and *F. rufa* did not differ in ecological dominance, they were both significantly less ecologically dominant than *T. sessile* (Table S5; Fig. S3)*. Formica rufa* was also significantly less ecologically dominant than *Myrmica* spp. and non-significantly less ecologically dominant than *F. podzolica* (Table S5; Fig. S3). *Formica podzolica* was significantly less ecologically dominant than *T. sessile*, but there were no significant pairwise differences between *F. podzolica* and *Myrmica* spp. or *T. sessile* and *Myrmica* spp. (Table S5; Fig. S3).

**Table S5.** Results from Tukey tests for multiple comparisons for differences in ecological dominance scores among ant taxa.

| Taxon pair | *z* score | *p*-value |
| --- | --- | --- |
| *Camponotus* spp. – *F. rufa* | 0.58 | 0.977 |
| *Camponotus* spp. – *Myrmica* spp. | -2.09 | 0.218 |
| *Camponotus* spp. – *F. podzolica* | -1.03 | 0.838 |
| *Camponotus* spp. – *T. sessile* | -3.29 | 0.009 |
| *F. rufa* – *Myrmica* spp. | -4.14 | <0.001 |
| *F. rufa* – *F. podzolica* | -2.53 | 0.080 |
| *F. rufa* – *T. sessile* | -5.97 | <0.001 |
| *Myrmica* spp. – *F. podzolica* | 1.71 | 0.415 |
| *Myrmica* spp. – *T. sessile* | -1.93 | 0.294 |
| *F. podzolica – T. sessile* | -3.63 | 0.003 |


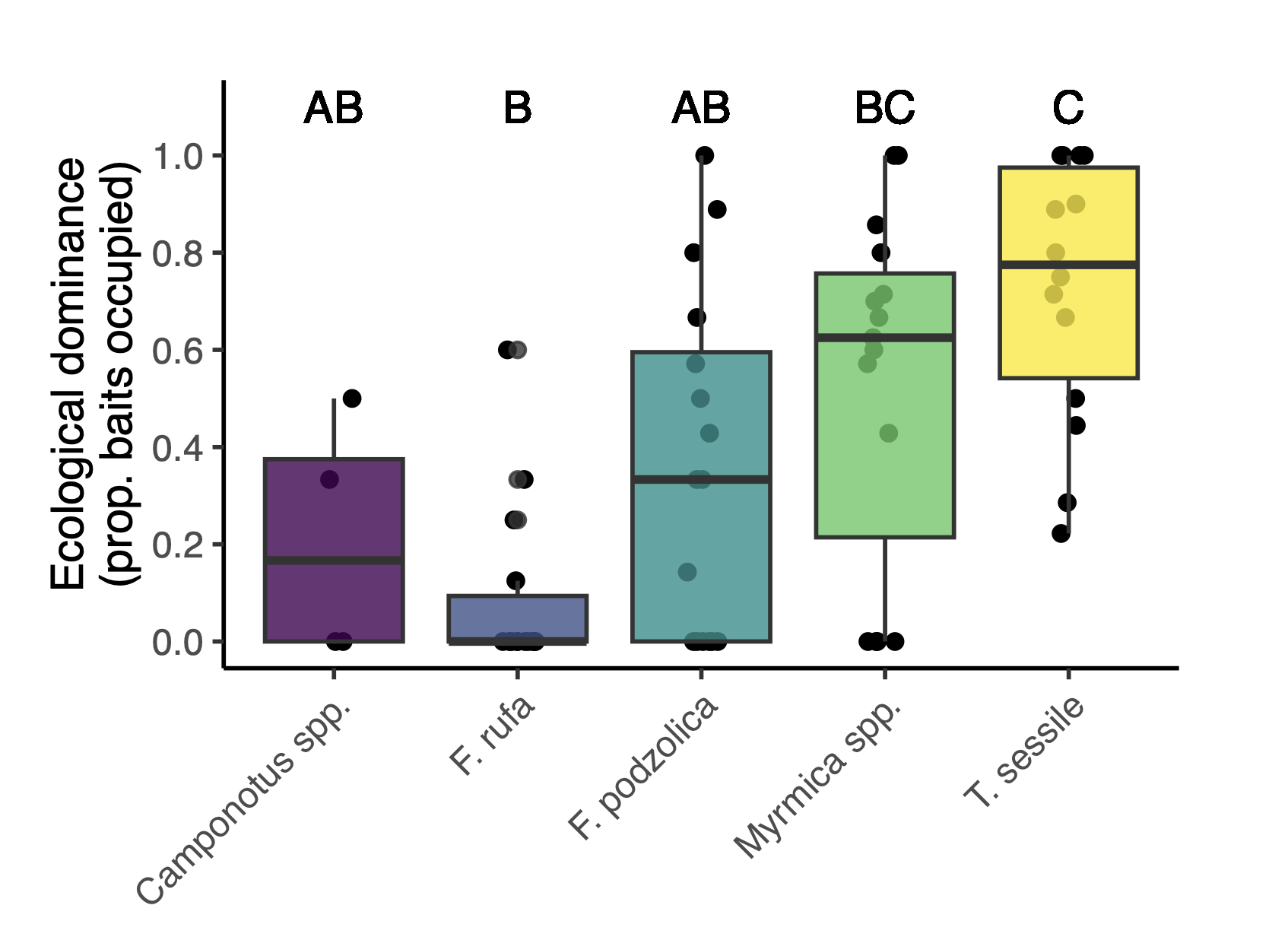


**Fig. S3.** Boxplots of ant ecological dominance, measured as the proportion of baits occupied out of all the stations where that taxon occurred in the pitfall traps (y-axis), of each of the five taxa (x-axis).

Correlations between dominance metrics

**Table S6.** Results from separate tests for correlations between dominance metrics.

| Dominance metrics | *t*-statistic | df | *p*-value | r |
| --- | --- | --- | --- | --- |
| Individual behavioral, colony behavioral | 3.86 | 3 | 0.031 | 0.91 |
| Individual behavioral, numerical in pitfalls | -2.88 | 3 | 0.064 | -0.86 |
| Individual behavioral, numerical in baits | -2.27 | 3 | 0.108 | -0.80 |
| Colony behavioral, numerical in pitfalls | -6.03 | 3 | 0.009 | -0.96 |
| Colony behavioral, numerical in baits | -2.19 | 3 | 0.116 | -0.78 |
| Individual behavioral, ecological | -1.72 | 3 | 0.184 | -0.70 |
| Colony behavioral, ecological | -1.43 | 3 | 0.248 | -0.64 |
| Numerical in pitfalls, numerical in baits | 3.01 | 3 | 0.057 | 0.87 |
| Numerical in pitfalls, ecological | 1.84 | 3 | 0.164 | 0.73 |
| Numerical in baits, ecological | 7.22 | 3 | 0.005 | 0.97 |

Discovery time

We tested for differences in resource discovery ability (speed at which baits were discovered) among ants with an LMER. The model included ant taxon as a fixed effect, and to account for differences in sampling effort across sites, we included the number of hours that baits were deployed as a covariate and site as a random effect. Because multiple ant taxa sometimes discovered the same baits, we also included bait nested within site as a random effect. There were significant differences among taxa in discovery ability (LMER: *F*_4,174_ = 4.51, *p* = 0.002; Fig. S4) regardless of sampling effort (LMER: *F*_1,16_ = 1.66, *p* = 0.217). To determine which ants differed, we conducted Tukey post-hoc tests. We found that *Camponotus* spp. discovered baits significantly more slowly than *Myrmica* spp. and non-significantly more slowly than *F. podzolica* and *T. sessile* (Table S7; Fig. S4). Similarly, *F. rufa* discovered baits significantly more slowly than *Myrmica* spp. (Table S7; Fig. S4). There were no significant differences in discovery time between any other taxa (Table S7; Fig. S4).

**Table S7.** Results from Tukey tests for multiple comparisons for differences in discovery ability among ant taxa.

| Taxon pair | *z* score | *p*-value |
| --- | --- | --- |
| *Camponotus* spp. – *F. rufa* | -0.87 | 0.896 |
| *Camponotus* spp. – *Myrmica* spp. | -3.06 | 0.016 |
| *Camponotus* spp. – *F. podzolica* | -2.59 | 0.060 |
| *Camponotus* spp. – *T. sessile* | -2.47 | 0.082 |
| *F. rufa* – *Myrmica* spp. | -2.99 | 0.020 |
| *F. rufa* – *F. podzolica* | -2.37 | 0.106 |
| *F. rufa* – *T. sessile* | -2.16 | 0.170 |
| *Myrmica* spp. – *F. podzolica* | 0.97 | 0.850 |
| *Myrmica* spp. – *T. sessile* | 1.86 | 0.304 |
| *F. podzolica – T. sessile* | 0.56 | 0.977 |


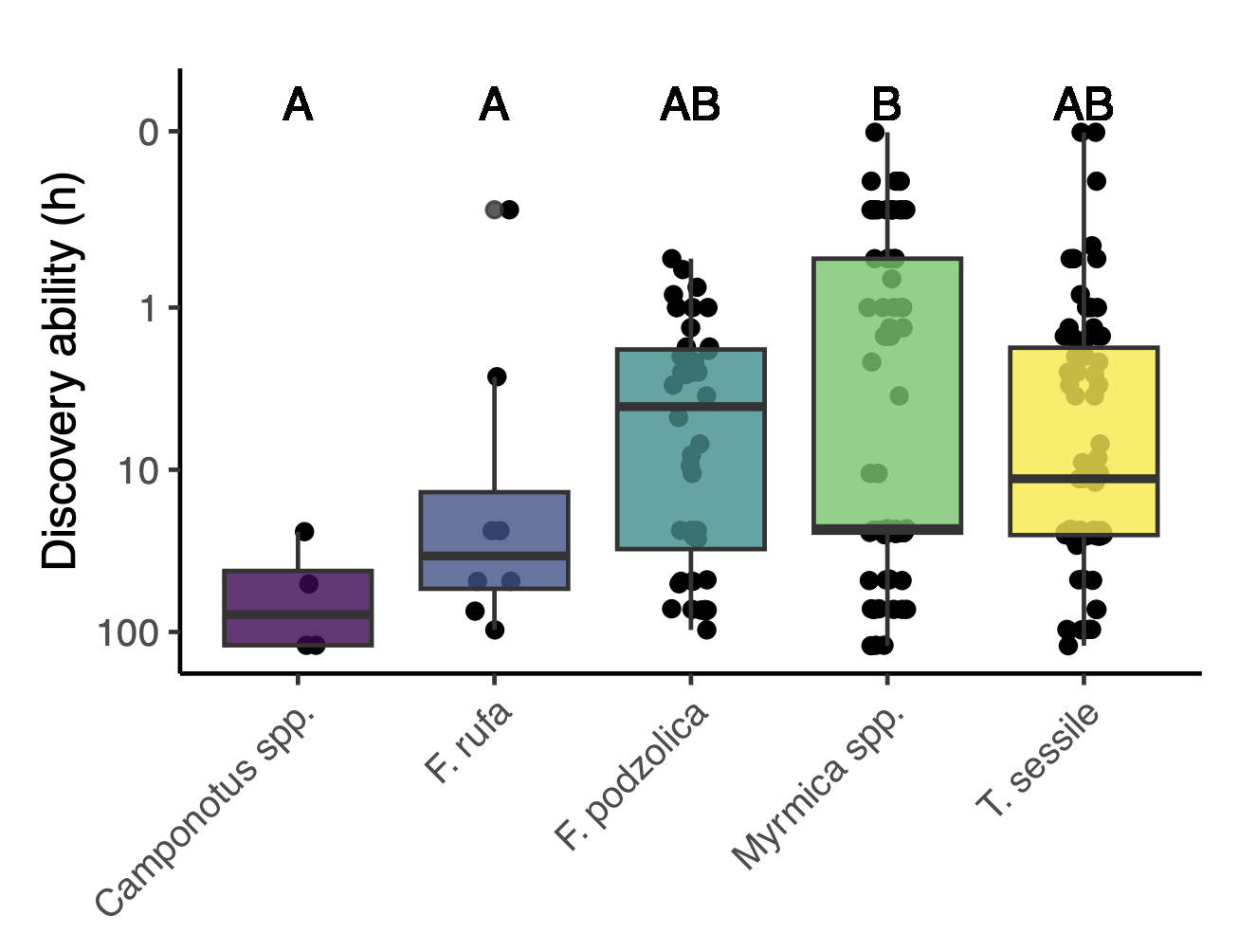


**Fig. S4.** Boxplots of ant resource discovery ability, measured as the time (number of hours; y-axis) at which each taxon (x-axis) was first observed at each 20% honey bait where it occurred. Note that the y-axis is reversed, such that faster resource discoverers (i.e., ants that took fewer hours to discover resources) are shown higher on the y-axis.

Ant traits hypothesized to underlie the dominance-discovery trade-off

We tested for differences among ants in traits hypothesized to underlie the dominance-discovery trade-off using separate linear models (LMs) constructed using the ‘lm()’ function in the ‘stats’ package (R Core Team 2020) and LMERs constructed using the ‘lmer()’ function in the ‘lme4’ package (Bates et al. 2015). To test for differences in ant head width, we constructed an LMER with ant taxon as a fixed effect and site as well as pitfall trap within site as random effects. To test for differences in forager body mass (*ln-*transformed to improve normality of residuals), we constructed a LM with ant taxon as the fixed effect. To test for differences in forager recruitment rate and the biomass of recruited foragers (both response variables *ln*-transformed to improve normality of residuals), we constructed separate LMERs. Both models included ant taxon as a fixed effect, and to account for differences in sampling effort across sites, we included the number of hours that baits were deployed as a covariate and site as a random effect. Because multiple ant taxa sometimes recruited to the same baits, we also included bait nested within site as a random effect.

There were significant differences among ant taxa in head width (LM: *F*_1,967_ = 937.44, *p* < 0.001). Post-hoc tests revealed significant differences among all taxa (Table S8). Ant taxa also differed in forager body mass (LM: *F*_4,76_ = 186.39, *p* < 0.001). There were significant differences among all taxa, except for between *Camponotus* spp. and *F. rufa* (Table S8). Moreover, taxa differed in forager recruitment rate (LMER: *F*_4,185_ = 10.69, *p* < 0.001). *Formica podzolica* recruited significantly fewer foragers to baits than *Myrmica* spp. and *T. sessile*, and *F. rufa* recruited fewer foragers than *Myrmica* spp. and *T. sessile* (Table S8). However, there were no detectable differences in recruitment rate between any other taxa (Table S8). Finally, ants significantly differed in the mean biomass of foragers recruited to baits (LMER: *F*_4,185_ = 55.11, *p* < 0.001). The biomass of recruits significantly differed among all taxa, except for between *F. rufa* and *F. podzolica* as well as between *F. podzolica* and *Myrmica* spp. (Table S8).

**Table S8.** Results from Tukey tests for multiple comparisons for differences in (a) forager head width, (b) forager body mass, (c) forager recruitment rate to baits, and (d) biomass of recruited foragers.

| Taxon pair | Test statistic | *p*-value |
| --- | --- | --- |
| (a) Forager head width | *z* score |  |
| *Camponotus* spp. – *F. rufa* | 12.17 | <0.001 |
| *Camponotus* spp. – *Myrmica* spp. | 22.56 | <0.001 |
| *Camponotus* spp. – *F. podzolica* | 20.08 | <0.001 |
| *Camponotus* spp. – *T. sessile* | 29.65 | <0.001 |
| *F. rufa* – *Myrmica* spp. | 30.63 | <0.001 |
| *F. rufa* – *F. podzolica* | 23.16 | <0.001 |
| *F. rufa* – *T. sessile* | 52.56 | <0.001 |
| *Myrmica* spp. – *F. podzolica* | -6.98 | <0.001 |
| *Myrmica* spp. – *T. sessile* | 28.62 | <0.001 |
| *F. podzolica – T. sessile* | 32.16 | <0.001 |
|  |  |  |
| (b) Forager body mass | *t* score |  |
| *Camponotus* spp. – *F. rufa* | 2.30 | 0.149 |
| *Camponotus* spp. – *Myrmica* spp. | 10.98 | <0.001 |
| *Camponotus* spp. – *F. podzolica* | 4.26 | <0.001 |
| *Camponotus* spp. – *T. sessile* | 17.31 | <0.001 |
| *F. rufa* – *Myrmica* spp. | 14.34 | <0.001 |
| *F. rufa* – *F. podzolica* | 3.21 | 0.016 |
| *F. rufa* – *T. sessile* | 23.76 | <0.001 |
| *Myrmica* spp. – *F. podzolica* | -9.90 | <0.001 |
| *Myrmica* spp. – *T. sessile* | 10.26 | <0.001 |
| *F. podzolica – T. sessile* | 18.92 | <0.001 |
|  |  |  |
| (c) Forager recruitment rate | *z* score |  |
| *Camponotus* spp. – *F. rufa* | 1.83 | 0.325 |
| *Camponotus* spp. – *Myrmica* spp. | 0.35 | 0.996 |
| *Camponotus* spp. – *F. podzolica* | 2.32 | 0.120 |
| *Camponotus* spp. – *T. sessile* | 0.22 | 0.999 |
| *F. rufa* – *Myrmica* spp. | -2.66 | 0.051 |
| *F. rufa* – *F. podzolica* | 0.33 | 0.997 |
| *F. rufa* – *T. sessile* | -2.85 | 0.029 |
| *Myrmica* spp. – *F. podzolica* | 5.36 | <0.001 |
| *Myrmica* spp. – *T. sessile* | -0.41 | 0.993 |
| *F. podzolica – T. sessile* | -5.97 | <0.001 |
|  |  |  |
| (d) Forager biomass | *z* score |  |
| *Camponotus* spp. – *F. rufa* | 2.72 | 0.043 |
| *Camponotus* spp. – *Myrmica* spp. | 5.46 | <0.001 |
| *Camponotus* spp. – *F. podzolica* | 4.29 | <0.001 |
| *Camponotus* spp. – *T. sessile* | 8.46 | <0.001 |
| *F. rufa* – *Myrmica* spp. | 3.23 | 0.009 |
| *F. rufa* – *F. podzolica* | 1.70 | 0.399 |
| *F. rufa* – *T. sessile* | 7.70 | <0.001 |
| *Myrmica* spp. – *F. podzolica* | -2.64 | 0.054 |
| *Myrmica* spp. – *T. sessile* | 9.77 | <0.001 |
| *F. podzolica – T. sessile* | 11.09 | <0.001 |

**
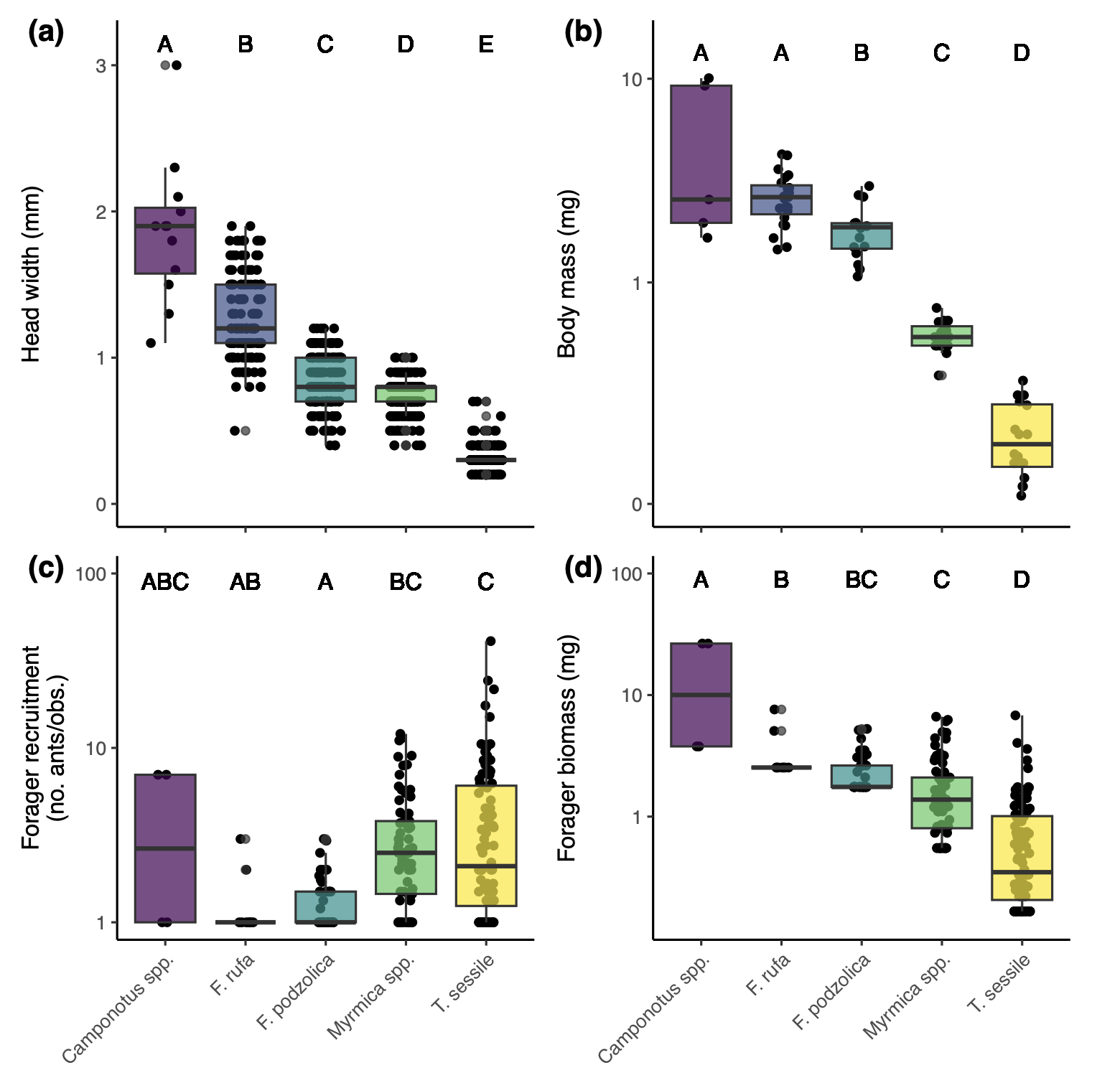
**

**Fig. S5.** Boxplots of ant traits hypothesized to underlie the dominance-discovery trade-off, including (a) forager head width (mm), (b) forager body mass (mg), (c) forager recruitment rate (mean number of ants per observation of a bait), and (d) the mean biomass of recruited foragers (mg; calculated as body mass x forager recruitment rate).

**Section S3. Principal component analysis of behavioral dominance and discovery ability**

**Fig. S6.** The relationship between PC1 and (a) individual-level behavioral dominance and (b) discovery ability. PC1 explained 96.7% of the covariation in behavioral dominance and discovery ability.

**Section S4. Meta-analysis**

To stabilize the variance among effect sizes prior to meta-analysis, each Spearman’s rank correlation coefficient was transformed using Fisher’s *Z*-transformation following the formula:

$${ES}_{Z}=0.5*ln\frac{1+r_{s}}{1-r_{s}}$$

where the effect size is *ES_Z_* and *r_s_* is the Spearman’s rank correlation coefficient. The variance weight was calculated as:

$$\frac{1}{n-3}$$

where *n* is the sample size, or number of species upon with the correlation was based.

**Table S9.** Summary of studies measuring the correlation (*r_s_*) between dominance and discovery ability among ant species within an ecological community.

|  |  |  | |  |  | Effect | |
| --- | --- | --- | --- | --- | --- | --- | --- |
| Dominance type | Discovery metric | Ref no. | Longitude | Latitude | No. spp. | *r_s_* | -, ns, or + |
| Behavioral (colony-level dominance in baits) | No./prop. baits discovered first | ^1^ | -109.2333 | 31.8667 | 7 | -0.79 | - |
|  | No./prop. baits discovered first, out of all baits where present | ^2^ | -48.3833 | -19.1667 | 16 | 0.58 | + |
|  |  | ^3^ | -76.935 | 38.986 | 8 | -0.83 | - |
|  |  | ^4^ | -43.9 | -21.7 | 9 | 0.30 | ns |
|  |  | ^5^ | 11.3 | 43.8 | 7 | 0.89 | + |
|  |  | ^6^ | -81.352 | 27.181 | 10 | 0.06 | ns |
|  |  | ^6^ | -81.352 | 27.181 | 11 | 0.46 | ns |
|  | Time to discover/recruit to baits | ^7^ | -57.0167 | -19.5667 | 9 | 0.07 | ns |
|  |  | ^7^ | -56.4667 | -21.1333 | 8 | 0.26 | ns |
|  |  | ^7^ | -47.1 | -22.8167 | 10 | -0.33 | ns |
|  |  | ^8^ | -79.9833 | 35.8667 | 6 | 0.09 | ns |
|  |  | ^9^ | 151.05 | -33.515 | 7 | 0.66 | ns |
|  |  | ^10^ | -122 | 38.5 | 7 | -0.79 | - |
|  |  | ^11^ | -106.99 | 38.96 | 5 | -0.63 | ns |
|  | Residuals of the number of baits discovered, regressed against abundance in traps | ^12^ | -83.5 | 35 | 7 | 0.00 | ns |
| Numerical | No./prop. baits discovered first | ^13^ | -95.0667 | 18.5667 | 12 | 0.661 | + |
|  |  | ^14^ | -171.5833 | -9.75 | 6 | 0.500 | ns |
|  |  | ^15^ | -64.135 | -28.326 | 5 | 0.526 | ns |
|  | No./prop. baits discovered first, out of all baits where present | ^16^ | 151.1666 | -34.1333 | 10 | 0.5 | ns |
|  |  | ^16^ | 151.1667 | -34.1333 | 11 | 0.589 | ns |
|  |  | ^16^ | -1.3333 | 51.7667 | 4 | 1 | + |
|  |  | ^16^ | 20.3667 | 64 | 6 | 0.812 | + |
|  |  | ^16^ | 20.3667 | 64 | 5 | 0.718 | ns |
|  |  | ^16^ | 31.5167 | -25.0167 | 9 | 0.784 | ns |
|  |  | ^16^ | 31.5167 | -25.0167 | 5 | 0.894 | + |
|  |  | ^16^ | 31.8 | -28.25 | 10 | 0.575 | ns |
|  |  | ^16^ | 31.8 | -28.25 | 7 | 0.929 | + |
|  | Time to discover/recruit to baits | ^11^ | -106.99 | 38.96 | 5 | 0.7 | ns |
| Ecological | No./prop. baits discovered first | ^15^ | -64.135 | -28.326 | 5 | 0.632 | ns |
|  | No./prop. baits discovered first, out of all baits where present | ^17^ | -76.2346 | 3.9166 | 15 | -0.76 | - |
|  |  | ^18^ | -58.9 | -31.2167 | 9 | 0.93 | - |
|  |  | ^19^ | 12.4 | 41.72 | 10 | 0.481 | ns |
|  |  | ^20^ | -49.2354 | -25.4467 | 12 | -0.17 | ns |
|  |  | ^21^ | 0.95 | 47.31 | 5 | -0.3 | ns |
|  |  | ^21^ | 0.95 | 47.31 | 4 | 0.33 | ns |
|  |  | ^21^ | -106.99 | 38.96 | 4 | 0.21 | ns |
|  |  | ^21^ | -52.65 | 5.17 | 14 | -0.22 | ns |
|  |  | ^21^ | 2.73 | 41.8 | 6 | -0.58 | ns |
|  |  | ^21^ | 2.83 | 41.99 | 4 | 0.06 | ns |
|  |  | ^21^ | 2.83 | 41.96 | 6 | -0.61 | ns |
|  |  | ^21^ | -3.54 | 36.82 | 5 | -0.39 | ns |
|  |  | ^21^ | 2.84 | 42 | 7 | 0.58 | ns |
|  |  | ^21^ | -3.51 | 36.91 | 8 | 0.05 | ns |
|  |  | ^21^ | -3.5 | 36.93 | 11 | 0.48 | ns |
|  |  | ^21^ | -3.5 | 36.95 | 9 | 0.13 | ns |
|  |  | ^21^ | -3.48 | 36.95 | 6 | 0.66 | ns |
|  |  | ^21^ | -3.48 | 36.96 | 5 | 0.73 | ns |
|  |  | ^21^ | -3.55 | 36.78 | 5 | 0.68 | ns |
|  |  | ^21^ | -3.81 | 40.77 | 4 | 0.77 | ns |
|  |  | ^21^ | -3.69 | 40.54 | 4 | -0.95 | - |
|  |  | ^21^ | -6.23 | 37.27 | 7 | 0.29 | ns |
|  |  | ^21^ | -2.17 | 47.22 | 5 | 0 | ns |
|  |  | ^21^ | -3.5 | 36.86 | 4 | -0.58 | ns |
|  | Time to discover/recruit to baits | ^11^ | -106.99 | 38.96 | 5 | 0.6 | ns |

1. **Lebrun and Feener 2007.** Behavioral dominance and discovery ability data were obtained from Table 1.
2. **Camarota et al. 2018.** Behavioral dominance and discovery ability data were obtained from Table 2.
3. **Fellers 1987.** Behavioral dominance scores were obtained from Table 8, and discovery abilities were obtained from Table 14.
4. **Sales et al. 2014.** Behavioral dominance scores were obtained from Table 2, and discovery abilities were extracted from Figure 3.
5. **Santini et al. 2007.** Behavioral dominance and discovery ability data were extracted from Figure 2c.
6. **Wiescher et al. 2011.** Behavioral dominance scores were obtained from Table 1, and discovery abilities were obtained from Table 2.
7. **Feener et al. 2008.** Discovery times were extracted from Figure 1, and dominance scores were extracted from Figure 3. Correlations between dominance and discovery are reported for all interactions (regardless of parasitoid presence) but separately for each of the three habitats included in the study.
8. **Stuble et al. 2013.** Behavioral dominance scores were obtained from Table 1, and discovery ability data were extracted from Supplemental Figure 1.
9. **Gibb and Hochuli 2004.** Behavioral dominance and discovery ability data were extracted from Figure 2.
10. **Holway 1999.** Behavioral dominance and discovery rankings were extracted from Figure 5.
11. **This study.** Behavioral, numerical, and ecological dominance as well as discovery ability scores are shown in Figure 3.
12. **Lessard et al. 2009.** Behavioral dominance and discovery ability data were extracted from Figure 1b.
13. **Antoniazzi et al. 2021.** Numerical dominance and discovery ability data were extracted from Figure 4.
14. **Sarty et al. 2006.** Numerical dominance data were obtained from Table 3, and discovery ability data were obtained from Table 2. Data were extracted from the bait card treatment only.
15. **Chifflet and Calcaterra 2025.** Numerical and ecological dominance and discovery ability data were extracted from Tables 2 and 3.
16. **Parr and Gibb 2012.** Spearman’s rank correlation coefficients were obtained from Table 2, and sample sizes were obtained by visually inspecting Figure 1.
17. **Achury et al. 2020.** Ecological dominance and discovery ability data were obtained from Table 4.
18. **Calcaterra et al. 2016.** Ecological dominance and discovery scores were extracted from Figure 3.
19. **Castracani et al. 2014.** Ecological dominance and discovery scores were extracted from Figure 1.
20. **Klunk and Pie 2021.** Ecological dominance and discovery ability data were extracted from Figure 1a.
21. **Sheard et al. 2020.** Spearman’s rank correlation coefficients were obtained from Supplementary Table S4.

**References**

Achury, R., P. Chacón de Ulloa, Á. Arcila, and A. V. Suarez. 2020. Habitat disturbance modifies dominance, coexistence, and competitive interactions in tropical ant communities. Ecological Entomology 45:1247–1262.

Antoniazzi, R., F. Camarota, M. Leponce, and W. Dáttilo. 2021. Discovery-defense strategy as a mechanism of social foraging of ants in tropical rainforest canopies. Behavioral Ecology 32:1022–1031.

Bates, D., M. Mächler, B. Bolker, and S. Walker. 2015. Fitting linear mixed-effects models using lme4. Journal of Statistical Software 67:1–48.

Calcaterra, L., S. Cabrera, and J. Briano. 2016. Local co-occurrence of several highly invasive ants in their native range: are they all ecologically dominant species? Insectes Sociaux 3:407–419.

Camarota, F., H. L. Vasconcelos, E. B. A. Koch, and S. Powell. 2018. Discovery and defense define the social foraging strategy of Neotropical arboreal ants. Behavioral Ecology and Sociobiology 72:110.

Castracani, C., F. A. Spotti, D. A. Grasso, A. Fanfani, and A. Mori. 2014. A new exception to the dominance-discovery trade-off rule in ant communities. Redia.

Chifflet, L., and L. A. Calcaterra. 2025. Dominance of a highly invasive ant is limited to the nesting territory of its supercolony. Ecological Entomology 50:150–162.

Feener, D. H., M. R. Orr, K. M. Wackford, J. M. Longo, W. W. Benson, and L. E. Gilbert. 2008. Geographic variation in resource dominance–discovery in Brazilian ant communities. Ecology 89:1824–1836.

Fellers, J. H. 1987. Interference and exploitation in a guild of woodland ants. Ecology 68:1466–1478.

Gibb, H., and D. F. Hochuli. 2004. Removal Experiment Reveals Limited Effects of a Behaviorally Dominant Species on Ant Assemblages. Ecology 85:648–657.

Hervé, M. R. 2022. RVAideMemoire: Testing and Plotting Procedures for Biostatistics. R package version 0.9-81-2:https://CRAN.R-project.org/package=RVAideMemoire.

Holway, D. A. 1999. Competitive mechanisms underlying the displacement of native ants by the invasive Argentine ant. Ecology 80:238–251.

Hothorn, T., F. Bretz, and P. Westfall. 2008. Simultaneous inference in general parametric models. Biometrical Journal 50:346–363.

Klunk, C. L., and M. R. Pie. 2021. No evidence for dominance–discovery trade-offs in Pheidole (Hymenoptera: Formicidae) assemblages. Canadian Journal of Zoology 99:1002–1008.

Lebrun, E. G., and D. H. F. Feener. 2007. When trade-offs interact: balance of terror enforces dominance discovery trade-off in a local ant assemblage. Journal of Animal Ecology 76:58–64.

Lessard, J.-P., R. R. Dunn, and N. J. Sanders. 2009. Temperature-mediated coexistence in temperate forest ant communities. Insectes Sociaux 56:149–156.

Parr, C. L., and H. Gibb. 2012. The discovery–dominance trade-off is the exception, rather than the rule. Journal of Animal Ecology 81:233–241.

R Core Team. 2020. R: A language and environment for statistical computing. R Foundation for Statistical Computing, Vienna, Austria. URL https://www.R-project.org/.

Sales, T. A., I. N. Hastenreiter, L. F. Ribeiro, and J. F. S. Lopes. 2014. Competitive Interactions in Ant Assemblage in a Rocky Field Environment: Is Being Fast and Attacking the Best Strategy? Sociobiology 61:258–264.

Santini, G., L. Tucci, L. Ottonetti, and Filippo Frizzi. 2007. Competition trade-offs in the organisation of a Mediterranean ant assemblage. Ecological Entomology 32:319–326.

Sarty, M., K. L. Abbott, and P. J. Lester. 2006. Habitat complexity facilitates coexistence in a tropical ant community. Oecologia 149:465–473.

Sheard, J. K., A. S. Nelson, J. D. Berggreen, R. Boulay, R. R. Dunn, and N. J. Sanders. 2020. Testing trade-offs and the dominance–impoverishment rule among ant communities. Journal of Biogeography n/a.

Stuble, K. L., M. A. Rodriguez-Cabal, G. L. McCormick, I. Jurić, R. R. Dunn, and N. J. Sanders. 2013. Tradeoffs, competition, and coexistence in eastern deciduous forest ant communities. Oecologia 171:981–992.

Wiescher, P. T., J. M. C. Pearce-Duvet, and D. H. Feener. 2011. Environmental context alters ecological trade-offs controlling ant coexistence in a spatially heterogeneous region. Ecological Entomology 36:549–559.
